# Breast cancer risks associated with missense variants in breast cancer susceptibility genes

**DOI:** 10.1186/s13073-022-01052-8

**Published:** 2022-05-18

**Authors:** Leila Dorling, Sara Carvalho, Jamie Allen, Michael T. Parsons, Cristina Fortuno, Anna González-Neira, Stephan M. Heijl, Muriel A. Adank, Thomas U. Ahearn, Irene L. Andrulis, Päivi Auvinen, Heiko Becher, Matthias W. Beckmann, Sabine Behrens, Marina Bermisheva, Natalia V. Bogdanova, Stig E. Bojesen, Manjeet K. Bolla, Michael Bremer, Ignacio Briceno, Nicola J. Camp, Archie Campbell, Jose E. Castelao, Jenny Chang-Claude, Stephen J. Chanock, Georgia Chenevix-Trench, NBCS Collaborators, J. Margriet Collée, Kamila Czene, Joe Dennis, Thilo Dörk, Mikael Eriksson, D. Gareth Evans, Peter A. Fasching, Jonine Figueroa, Henrik Flyger, Marike Gabrielson, Manuela Gago-Dominguez, Montserrat García-Closas, Graham G. Giles, Gord Glendon, Pascal Guénel, Melanie Gündert, Andreas Hadjisavvas, Eric Hahnen, Per Hall, Ute Hamann, Elaine F. Harkness, Mikael Hartman, Frans B. L. Hogervorst, Antoinette Hollestelle, Reiner Hoppe, Anthony Howell, kConFab Investigators, SGBCC Investigators, Anna Jakubowska, Audrey Jung, Elza Khusnutdinova, Sung-Won Kim, Yon-Dschun Ko, Vessela N. Kristensen, Inge M. M. Lakeman, Jingmei Li, Annika Lindblom, Maria A. Loizidou, Artitaya Lophatananon, Jan Lubiński, Craig Luccarini, Michael J. Madsen, Arto Mannermaa, Mehdi Manoochehri, Sara Margolin, Dimitrios Mavroudis, Roger L. Milne, Nur Aishah Mohd Taib, Kenneth Muir, Heli Nevanlinna, William G. Newman, Jan C. Oosterwijk, Sue K. Park, Paolo Peterlongo, Paolo Radice, Emmanouil Saloustros, Elinor J. Sawyer, Rita K. Schmutzler, Mitul Shah, Xueling Sim, Melissa C. Southey, Harald Surowy, Maija Suvanto, Ian Tomlinson, Diana Torres, Thérèse Truong, Christi J. van Asperen, Regina Waltes, Qin Wang, Xiaohong R. Yang, Paul D. P. Pharoah, Marjanka K. Schmidt, Javier Benitez, Bas Vroling, Alison M. Dunning, Soo Hwang Teo, Anders Kvist, Miguel de la Hoya, Peter Devilee, Amanda B. Spurdle, Maaike P. G. Vreeswijk, Douglas F. Easton

**Affiliations:** 1 Centre for Cancer Genetic Epidemiology, Department of Public Health and Primary Care, University of Cambridge, Cambridge, CB1 8RN UK; 2 Department of Genetics and Computational Biology, QIMR Berghofer Medical Research Institute, Brisbane, QLD 4006 Australia; 3 Human Cancer Genetics Programme, Spanish National Cancer Research Centre (CNIO), 28029 Madrid, Spain; 4 Bio-Prodict, Nijmegen, The Netherlands; 5 Family Cancer Clinic, The Netherlands Cancer Institute - Antoni Van Leeuwenhoek Hospital, Amsterdam, 1066 CX The Netherlands; 6 Division of Cancer Epidemiology and Genetics, Department of Health and Human Services, National Cancer Institute, National Institutes of Health, Bethesda, MD 20850 USA; 7 Fred A. Litwin Center for Cancer Genetics, Lunenfeld-Tanenbaum Research Institute of Mount Sinai Hospital, Toronto, ON M5G 1X5 Canada; 8 Department of Molecular Genetics, University of Toronto, Toronto, ON M5S 1A8 Canada; 9 Translational Cancer Research Area, University of Eastern Finland, 70210 Kuopio, Finland; 10 Institute of Clinical Medicine, Oncology, University of Eastern Finland, 70210 Kuopio, Finland; 11 Department of Oncology, Cancer Center, Kuopio University Hospital, 70210 Kuopio, Finland; 12 Institute of Medical Biometry and Epidemiology, University Medical Center Hamburg-Eppendorf, 20246 Hamburg, Germany; 13 Department of Gynecology and Obstetrics, Comprehensive Cancer Center Erlangen-EMN, University Hospital Erlangen, Friedrich-Alexander University Erlangen-Nuremberg (FAU), 91054 Erlangen, Germany; 14 Division of Cancer Epidemiology, German Cancer Research Center (DKFZ), 69120 Heidelberg, Germany; 15 Institute of Biochemistry and Genetics, Ufa Federal Research Centre of the Russian Academy of Sciences, Ufa, 450054 Russia; 16 Department of Radiation Oncology, Hannover Medical School, 30625 Hannover, Germany; 17 Gynaecology Research Unit, Hannover Medical School, 30625 Hannover, Germany; 18 N.N. Alexandrov Research Institute of Oncology and Medical Radiology, 223040 Minsk, Belarus; 19 Copenhagen General Population Study, Herlev and Gentofte Hospital, Copenhagen University Hospital, 2730 Herlev, Denmark; 20 Department of Clinical Biochemistry, Herlev and Gentofte Hospital, Copenhagen University Hospital, 2730 Herlev, Denmark; 21 Faculty of Health and Medical Sciences, University of Copenhagen, 2200 Copenhagen, Denmark; 22 Medical Faculty, Universidad de La Sabana, 140013 Bogota, Colombia; 23 Department of Internal Medicine and Huntsman Cancer Institute, University of Utah, Salt Lake City, UT 84112 USA; 24 Centre for Genomic and Experimental Medicine, Institute of Genetics & Cancer, The University of Edinburgh, Western General Hospital, Edinburgh, EH4 2XU UK; 25 Usher Institute of Population Health Sciences and Informatics, The University of Edinburgh, Edinburgh, EH16 4UX UK; 26 Oncology and Genetics Unit, Instituto de Investigacion Sanitaria Galicia Sur (IISGS), Xerencia de Xestion Integrada de Vigo-SERGAS, 36312 Vigo, Spain; 27 Cancer Epidemiology Group, University Cancer Center Hamburg (UCCH), University Medical Center Hamburg-Eppendorf, 20246 Hamburg, Germany; 28 Department of Cancer Genetics, Institute for Cancer Research, Oslo University Hospital-Radiumhospitalet, 0379 Oslo, Norway; 29 Institute of Clinical Medicine, Faculty of Medicine, University of Oslo, 0450 Oslo, Norway; 30 Department of Research, Vestre Viken Hospital, 3019 Drammen, Norway; 31 Department of Tumor Biology, Institute for Cancer Research, Oslo University Hospital-Radiumhospitalet, 0379 Oslo, Norway; 32 Department of Oncology, Division of Surgery, Cancer and Transplantation Medicine, Oslo University Hospital-Radiumhospitalet, 0379 Oslo, Norway; 33 Department of Oncology, Akershus University Hospital, 1478 Lørenskog, Norway; 34 Breast Cancer Research Consortium, Oslo University Hospital, 0379 Oslo, Norway; 35 Department of Medical Genetics, Oslo University Hospital and University of Oslo, 0379 Oslo, Norway; 36 Department of Community Medicine, The Arctic University of Norway, 9037 Tromsø, Norway; 37 Department of Clinical Genetics, Erasmus University Medical Center, Rotterdam, 3015 CN The Netherlands; 38 Department of Medical Epidemiology and Biostatistics, Karolinska Institutet, 171 65 Stockholm, Sweden; 39 Division of Evolution and Genomic Sciences, School of Biological Sciences, Faculty of Biology, Medicine and Health, University of Manchester, Manchester Academic Health Science Centre, Manchester, M13 9WL UK; 40 North West Genomics Laboratory Hub, Manchester Centre for Genomic Medicine, St Mary’s Hospital, Manchester University NHS Foundation Trust, Manchester Academic Health Science Centre, Manchester, M13 9WL UK; 41 Nightingale & Genesis Prevention Centre, Wythenshawe Hospital, Manchester University NHS Foundation Trust, Manchester, M23 9LT UK; 42 NIHR Manchester Biomedical Research Centre, Manchester University NHS Foundation Trust, Manchester Academic Health Science Centre, Manchester, M13 9WL UK; 43 David Geffen School of Medicine, Department of Medicine Division of Hematology and Oncology, University of California at Los Angeles, Los Angeles, CA 90095 USA; 44 Cancer Research UK Edinburgh Centre, The University of Edinburgh, Edinburgh, EH4 2XR UK; 45 Department of Breast Surgery, Herlev and Gentofte Hospital, Copenhagen University Hospital, 2730 Herlev, Denmark; 46 Fundación Pública Galega de Medicina Xenómica, Instituto de Investigación Sanitaria de Santiago de Compostela (IDIS), Complejo Hospitalario Universitario de Santiago, SERGAS, , 15706 Santiago de Compostela, Spain; 47 Moores Cancer Center, University of California San Diego, La Jolla, CA 92037 USA; 48 Cancer Epidemiology Division, Cancer Council Victoria, Melbourne, VIC 3004 Australia; 49 Centre for Epidemiology and Biostatistics, Melbourne School of Population and Global Health, The University of Melbourne, Melbourne, VIC 3010 Australia; 50 Precision Medicine, School of Clinical Sciences at Monash Health, Monash University, Clayton, VIC 3168 Australia; 51 Team “Exposome and Heredity”, CESP, Inserm, Gustave Roussy, University Paris-Saclay, UVSQ, Villejuif, France; 52 Molecular Epidemiology Group, C080, German Cancer Research Center (DKFZ), 69120 Heidelberg, Germany; 53 Molecular Biology of Breast Cancer, University Womens Clinic Heidelberg, University of Heidelberg, 69120 Heidelberg, Germany; 54 Institute of Diabetes Research, Helmholtz Zentrum München, German Research Center for Environmental Health, 85764 Neuherberg, Germany; 55 Cancer Genetics, Therapeutics and Ultrastructural Pathology, The Cyprus Institute of Neurology & Genetics, 2371 Nicosia, Cyprus; 56 Cyprus School of Molecular Medicine, The Cyprus Institute of Neurology & Genetics, 2371 Nicosia, Cyprus; 57 Center for Familial Breast and Ovarian Cancer, Faculty of Medicine and University Hospital Cologne, University of Cologne, 50937 Cologne, Germany; 58 Center for Integrated Oncology (CIO), Faculty of Medicine, University Hospital Cologne, University of Cologne, 50937 Cologne, Germany; 59 Department of Oncology, 118 83 Södersjukhuset, Stockholm, Sweden; 60 Molecular Genetics of Breast Cancer, German Cancer Research Center (DKFZ), 69120 Heidelberg, Germany; 61 Division of Informatics, Imaging and Data Sciences, Faculty of Biology, Medicine and Health, University of Manchester, Manchester Academic Health Science Centre, Manchester, M13 9PT UK; 62 Saw Swee Hock School of Public Health, National University of Singapore and National University Health System, Singapore, 117549 Singapore; 63 Department of Surgery, National University Health System, Singapore, 119228 Singapore; 64 Department of Surgery, Yong Loo Lin School of Medicine, National University of Singapore and National University Health System, Singapore, 119228 Singapore; 65 Department of Medical Oncology, Erasmus MC Cancer Institute, Rotterdam, 3015 GD The Netherlands; 66 Dr. Margarete Fischer-Bosch-Institute of Clinical Pharmacology, 70376 Stuttgart, Germany; 67 University of Tübingen, 72074 Tübingen, Germany; 68 Division of Cancer Sciences, University of Manchester, Manchester, M13 9PL UK; 69 Research Department, Peter MacCallum Cancer Center, Melbourne, VIC 3000 Australia; 70 Department of Oncology, Sir Peter MacCallum, The University of Melbourne, Melbourne, VIC 3000 Australia; 71 Human Genetics Division, Genome Institute of Singapore, Singapore, 138672 Singapore; 72 Department of Medicine, Yong Loo Lin School of Medicine, National University of Singapore and National University Health System, Singapore, 119077 Singapore; 73 Cancer Genetics Service, National Cancer Centre, Singapore, 169610 Singapore; 74 Breast Department, KK Women’s and Children’s Hospital, Singapore, 229899 Singapore; 75 SingHealth Duke-NUS Breast Centre, Singapore, 168753 Singapore; 76 Department of General Surgery, Tan Tock Seng Hospital, Singapore, 308433 Singapore; 77 Division of Surgical Oncology, National Cancer Centre, Singapore, 169610 Singapore; 78 Department of General Surgery, Singapore General Hospital, Singapore, 169608 Singapore; 79 Division of Breast Surgery, Department of General Surgery, Changi General Hospital, Singapore, 529889 Singapore; 80 Division of Radiation Oncology, National Cancer Centre, Singapore, 169610 Singapore; 81 Division of Medical Oncology, National Cancer Centre, Singapore, 169610 Singapore; 82 Department of Genetics and Pathology, Pomeranian Medical University, 71-252 Szczecin, Poland; 83 Independent Laboratory of Molecular Biology and Genetic Diagnostics, Pomeranian Medical University, 71-252 Szczecin, Poland; 84 Department of Genetics and Fundamental Medicine, Bashkir State University, Ufa, 450000 Russia; 85 Department of Surgery, Daerim Saint Mary’s Hospital, Seoul, 07442 Korea; 86 Department of Internal Medicine, Johanniter GmbH Bonn, Johanniter Krankenhaus, 53113 Bonn, Germany; 87 Department of Human Genetics, Leiden University Medical Center, Leiden, 2333 ZA The Netherlands; 88 Department of Clinical Genetics, Leiden University Medical Center, Leiden, 2333 ZA The Netherlands; 89 Department of Molecular Medicine and Surgery, Karolinska Institutet, 171 76 Stockholm, Sweden; 90 Department of Clinical Genetics, Karolinska University Hospital, 171 76 Stockholm, Sweden; 91 Division of Population Health, Health Services Research and Primary Care, School of Health Sciences, Faculty of Biology, Medicine and Health, The University of Manchester, Manchester, M13 9PL UK; 92 Centre for Cancer Genetic Epidemiology, Department of Oncology, University of Cambridge, Cambridge, CB1 8RN UK; 93 Institute of Clinical Medicine, Pathology and Forensic Medicine, University of Eastern Finland, 70210 Kuopio, Finland; 94 Biobank of Eastern Finland, Kuopio University Hospital, Kuopio, Finland; 95 Department of Clinical Science and Education, Södersjukhuset, Karolinska Institutet, 118 83 Stockholm, Sweden; 96 Department of Medical Oncology, University Hospital of Heraklion, 711 10 Heraklion, Greece; 97 Breast Cancer Research Unit, Faculty of Medicine, University Malaya Cancer Research Institute, University of Malaya, 50603 Kuala Lumpur, Malaysia; 98 Department of Surgery, Faculty of Medicine, University of Malaya, 50603 Kuala Lumpur, Malaysia; 99 Department of Obstetrics and Gynecology, Helsinki University Hospital, University of Helsinki, 00290 Helsinki, Finland; 100 Department of Genetics, University Medical Center Groningen, University Groningen, Groningen, 9713 GZ The Netherlands; 101 Department of Preventive Medicine, Seoul National University College of Medicine, Seoul, 03080 Korea; 102 Convergence Graduate Program in Innovative Medical Science, Seoul National University College of Medicine, Seoul, 03080 South Korea; 103 Cancer Research Institute, Seoul National University, Seoul, 03080 Korea; 104 Genome Diagnostics Program, IFOM - the FIRC Institute of Molecular Oncology, 20139 Milan, Italy; 105 Unit of Molecular Bases of Genetic Risk and Genetic Testing, Department of Research, Fondazione IRCCS Istituto Nazionale Dei Tumori (INT), 20133 Milan, Italy; 106 Department of Oncology, University Hospital of Larissa, 411 10 Larissa, Greece; 107 School of Cancer & Pharmaceutical Sciences, Comprehensive Cancer Centre, Guy’s Campus, King’s College London, London, UK; 108 Center for Molecular Medicine Cologne (CMMC), Faculty of Medicine and University Hospital Cologne, University of Cologne, 50931 Cologne, Germany; 109 Department of Clinical Pathology, The University of Melbourne, Melbourne, VIC 3010 Australia; 110 Institute of Cancer and Genomic Sciences, University of Birmingham, Birmingham, B15 2TT UK; 111 Wellcome Trust Centre for Human Genetics and Oxford NIHR Biomedical Research Centre, University of Oxford, Oxford, OX3 7BN UK; 112 Institute of Human Genetics, Pontificia Universidad Javeriana, 110231 Bogota, Colombia; 113 Division of Molecular Pathology, The Netherlands Cancer Institute - Antoni Van Leeuwenhoek Hospital, Amsterdam, 1066 CX The Netherlands; 114 Division of Psychosocial Research and Epidemiology, The Netherlands Cancer Institute - Antoni Van Leeuwenhoek Hospital, Amsterdam, 1066 CX The Netherlands; 115 Biomedical Network On Rare Diseases (CIBERER), 28029 Madrid, Spain; 116 Centre for Molecular and Biomolecular Informatics (CMBI), Radboud University Medical Center, Nijmegen, The Netherlands; 117 Breast Cancer Research Programme, Cancer Research Malaysia, Subang Jaya, 47500 Selangor, Malaysia; 118 Division of Oncology and Pathology, Department of Clinical Sciences Lund, Lund University, 22381 Lund, Sweden; 119 Molecular Oncology Laboratory, Hospital Clinico San Carlos, IdISSC (Instituto de Investigación Sanitaria del Hospital Clínico San Carlos), 28040 Madrid, Spain; 120 Department of Pathology, Leiden University Medical Center, Leiden, 2333 ZA The Netherlands

**Keywords:** Breast cancer, Genetic epidemiology, Risk prediction, Missense variants

## Abstract

**Background:**

Protein truncating variants in *ATM*, *BRCA1*, *BRCA2*, *CHEK2*, and *PALB2* are associated with increased breast cancer risk, but risks associated with missense variants in these genes are uncertain.

**Methods:**

We analyzed data on 59,639 breast cancer cases and 53,165 controls from studies participating in the Breast Cancer Association Consortium BRIDGES project. We sampled training (80%) and validation (20%) sets to analyze rare missense variants in *ATM* (1146 training variants), *BRCA1* (644), *BRCA2* (1425),* CHEK2* (325), and *PALB2* (472). We evaluated breast cancer risks according to five in silico prediction-of-deleteriousness algorithms, functional protein domain, and frequency, using logistic regression models and also mixture models in which a subset of variants was assumed to be risk-associated.

**Results:**

The most predictive in silico algorithms were Helix (*BRCA1*, *BRCA2* and *CHEK2*) and CADD (*ATM*). Increased risks appeared restricted to functional protein domains for *ATM* (FAT and PIK domains) and *BRCA1* (RING and BRCT domains). For *ATM*, *BRCA1*, and *BRCA2*, data were compatible with small subsets (approximately 7%, 2%, and 0.6%, respectively) of rare missense variants giving similar risk to those of protein truncating variants in the same gene. For *CHEK2*, data were more consistent with a large fraction (approximately 60%) of rare missense variants giving a lower risk (OR 1.75, 95% CI (1.47–2.08)) than *CHEK2* protein truncating variants. There was little evidence for an association with risk for missense variants in *PALB2*. The best fitting models were well calibrated in the validation set.

**Conclusions:**

These results will inform risk prediction models and the selection of candidate variants for functional assays and could contribute to the clinical reporting of gene panel testing for breast cancer susceptibility.

**Supplementary Information:**

The online version contains supplementary material available at 10.1186/s13073-022-01052-8.

## Background

Genetic testing for cancer susceptibility is now part of mainstream clinical practice. For breast cancer susceptibility, genetic testing generally focuses on high-risk genes, notably *BRCA1*, *BRCA2*,* PALB2*, and *TP53*, but testing of larger panels that include so-called “moderate-risk” genes is being increasingly offered [1]. While the evidence that many of these genes are risk associated is clear, for most this evidence is based on carrying a protein truncating variant (PTV). Besides PTVs, genetic testing also identifies missense variants for which the impact on protein function and associated cancer risk is generally unknown (“variants of uncertain significance” (VUS)), resulting in a major problem for genetic counselling. Some missense variants have been shown to confer risk [2, 3] with risk estimates comparable to PTVs, and it is possible that missense variants contribute substantially to risk [4, 5], at least in some genes. However, defining the set of missense variants in each gene that may confer risk, and their associated risk estimates, presents an ongoing problem.

Resolving this problem is complex as most variants are individually very rare, so the evidence must be based on combining data across multiple variants in a statistical model. To this end, efforts have been made to develop statistical algorithms that score missense variants according to in silico features that may predict pathogenicity. Here, we have compared the usefulness of five in silico algorithms in predicting breast cancer risk associated with missense variants using sequenced germline DNA from more than 59,000 cases and 53,000 controls from studies in the Breast Cancer Association Consortium (BCAC) [6] participating in the BRIDGES project [7]. We used the most predictive in silico algorithm to estimate the risks of breast cancer associated with subsets of rare missense variants, defined by categories of the in silico score, in *ATM*, *BRCA1*, *BRCA2*, *CHEK2*, and *PALB2.* These predictions were then validated using an independent dataset.

## Methods

### Subjects

We included data from female breast cancer patients (cases) and unaffected controls from 44 studies participating in the BRIDGES project, as previously documented [7]. These studies are a subset of studies participating in the Breast Cancer Association Consortium (BCAC) for which targeted sequencing was performed using the BRIDGES panel (see below). Details of the participating studies, including the enrollment of cases and controls and sample sizes, are given in Additional File 1: Tables S1 and S2. Of these, 30 were population-based or hospital-based studies (hereafter: population studies) including cases and controls sampled independently of family history. A further 14 studies oversampled cases with a family history of breast cancer (hereafter: familial studies). All studies were approved by the relevant ethical review boards and used appropriate consent procedures. Five duplicated samples were identified and removed. After quality control procedures (see below), 53,165 controls and 59,639 cases with an invasive (53,838; 90.3%) or in situ (4,153; 7.0%) tumor, or tumor of unknown invasiveness (1648; 2.7%), were included in the analyses. Of these, 50,414 controls and 48,230 cases were from population studies.

### Laboratory methods, variant calling, and classification

The BRIDGES project performed targeted sequencing on a panel of 34 genes [7]. Of these five (*ATM*, *BRCA1*, *BRCA2*, *CHEK2*, *PALB2*) were chosen for further analysis and presented here. These five genes, where the evidence for association with breast cancer risk is strongest, are most relevant to risk prediction and included in the current version of the BOADICEA/CanRisk risk prediction tool [8]. Details of library preparation, sequencing, variant calling, quality control procedures, and variant classification has been documented previously [7]. Missense variants in the entire gene were identified using the Ensembl Variant Effect Predictor (VEP; version 101.0) [9]. Rare variants for in silico analysis were defined as those with allele frequency < 0.1% (calculated as previously described [7]); in addition, variants with frequency < 5% were retained for a frequency-based analysis. Carriers of missense variants predicted to affect RNA splicing, according to the MaxEntScan tool [10] and SpliceAI scores [11], were removed (see Additional File 2: Table S3). Variants were annotated for functional protein domain location, defined according to published literature, the UniProt Knowledgebase [12], and for *BRCA1* and BRCA2, the ENIGMA *BRCA1/2* expert panel guidelines [13] (see Additional File 1: Table S4). Variants were also classified for disease pathogenicity assertion in ClinVar [14] with a filter for no conflicting interpretations; for *BRCA1* and *BRCA2*, variants were also reviewed against the ENIGMA *BRCA1/2* expert panel guidelines. The ENIGMA terminology report [15] reserves use of the word “pathogenic” to describe variants associated with at least a twofold cancer risk; however, for the purpose of this article, we describe any variant associated with risk as pathogenic.

Variants were scored using five in silico prediction algorithms: Align-GVGD [16], Combined Annotation Dependent Depletion (CADD; version 1.4) [17], Rare Exome Variant Ensemble Learner (REVEL) [18], BayesDel (without allele frequency; version 1) [19], and Helix (version 4.2.0) [20]. The first four are widely used for variant classification in cancer susceptibility genes. Align-GVGD classifies variants according to the level of cross-species conservation observed for a single missense substitution while considering the biophysical characteristics of the amino acids. CADD, BayesDel, and REVEL are ensemble methods that integrate several different annotations, including conservation metrics, regulatory information, transcript information, and protein-level scores, into a single score of deleteriousness. Helix combines structural, alignment, and gene data with a strict training regime where circularity is actively avoided to produce a variant score and certainty estimate. All variants were scored using default software settings. For Align-GVGD, the sequence alignment with the deepest phylogeny level was used. Variants in *BRCA1* and *BRCA2* were also annotated with the predictions of Hart et al. [21], who developed two BRCA-specific in silico algorithms (Random Forest (RF) and Naïve Voting Method (NVM)) to classify missense variants as functionally damaging or neutral. In addition, *BRCA1* variants were annotated using the prediction of loss-of-function made by the Saturation Genome Editing (SGE) experiments of Findlay et al. [22], which involved a comprehensive functional assessment of missense variants lying within the functional domain coding regions of *BRCA1*. *BRCA2* variants were annotated using homology-directed DNA repair (HDR) assay scores and predictions of pathogenicity from Richardson et al. [23]. For *PALB2*, variants were annotated with five different assay scores measuring HDR activity, PARPi sensitivity, and homologous recombination (HR) efficiency from the functional screening studies of Boonen et al. [24], Rodrigue et al. [25] and Wiltshire et al. [26].

### Statistical analysis

The dataset was split into a training (80% of individuals) and a validation (20%) set. Samples for the validation set were selected randomly from population studies of cases unselected for family history of breast cancer and controls, in countries contributing a total of > 5000 samples (Denmark, Germany, Singapore (Chinese), Sweden, UK, USA). All remaining samples were included in the training set. The training set included 37,211 cases from population studies, 11,409 cases from familial studies, and 42,334 controls. Of these, 3818 individuals were carriers of PTVs in one or more of the five genes under consideration and were excluded from all analyses except the mixture models (see below). The validation set included 11,019 cases and 10,831 controls from population studies and did not include any carriers of PTVs. Oversampling of cases with a family history increases power but leads to biased effect sizes, so we chose this approach to maximize the power to discriminate between models in the training set, which could then be refit and tested on a dataset unselected for family history. All analyses were adjusted for country as a covariate; in addition, for Malaysia and Singapore, the three distinct ethnic groups (Chinese, Indian, Malay) were treated as different strata, and the UK was treated as three strata (SEARCH from East Anglia, GENSCOT from Scotland, and PROCAS and FHRISK from north-west England).

### Training dataset analysis

An analysis flow diagram is presented in Fig. S1 (see Additional File 1). Analyses were performed in R version 4.0.3 (R: A Language and Environment for Statistical Computing; http://www.r-project.org). We first used logistic regression (LR) to explore which of the five in silico scores (Align-GVGD, BayesDel, CADD, Helix, and REVEL—all analyzed as continuous variables) were most strongly associated with risk of breast cancer. In addition, to assess the utility of gene-specific in silico tools, we analyzed the Hart et al. RF and NVM in silico predictions for *BRCA1* and *BRCA2*. To evaluate the usefulness of functional predictions, we also analyzed the BRCA1 SGE score; the Richardson et al. BRCA2 HDR score; and, for *PALB2*, five functional assay scores. These analyses were restricted to carriers of a rare (frequency < 0.1%) missense variant in the training set, with an endpoint of breast cancer occurrence (yes/no). The strongest predictors were used to test the association of different categories of the score(s) compared to a baseline category, in conjunction with functional protein domains, and hence create a set of risk categories. LR was then used in the training set (carriers and non-carriers) to estimate the odds ratios (OR) associated with different risk categories. As an alternative approach, we fitted mixture models in which only a proportion of variants (α) was assumed to be risk associated in the given gene; the OR was assumed to be the same for all risk associated variants, but the proportion of risk associated variants varied by risk category (as defined in the LR models). This model is motivated by the binary variant classification approach used in clinical genetics, where all variants are assumed to be either associated with moderate-high risk (likely pathogenic) or not (likely benign) [27]. We considered two types of mixture model: a constrained model in which the missense OR was equal to that of PTVs, and an unconstrained model in which the missense OR could differ from the PTV OR. Carriers of PTVs in the gene under consideration were re-included in the mixture models (to allow the risk associated missense OR to be constrained to the PTV OR). The mixture models were fitted using an expectation–maximization (EM) algorithm [28]. In the expectation step, the (posterior) probability that each variant was risk associated, given the case control data on that variant in the training set and the current parameter values was calculated. These probabilities were then used as weights in a logistic regression analysis in the maximization step. In a case–control dataset, the naïve proportions, *α*, will be biased because risk associated variants are more likely to be found in cases. For the final models, therefore, we also computed the proportions based only on variants reported in controls. To evaluate the overall fit of the models, we compared log-likelihoods.

The initial model selection was based on all samples, but final parameter estimates were obtained from population studies only. In the results, the ORs, *P*-values, and *α* presented are from population studies, unless indicated by the suffix “ALL”.

Case-only analyses of age at diagnosis, with risk category as the outcome variable, were performed to evaluate trends in the ORs for variant risk category by age. We evaluated individual risk variants previously reported in literature and, in aggregate, those classified as “pathogenic” or “likely pathogenic” (hereafter, all termed: (likely) pathogenic) according to clinical guidelines. To examine whether rare variant frequency is associated with risk, we used a carrier-only LR analysis to test frequency up to 0.5% on a continuous scale and a log scale, and to compare rare variants in two groups: frequency < 0.1% versus frequency 0.1–0.5%. We also performed burden analyses within each gene comparing the risk for non-carriers to the risk for carriers of variants in one of four frequency groups: < 0.1%; 0.1–0.5%; 0.5–1%; and 1–5%. Variants with frequency between 0.1 and 5% were also evaluated individually.

### Validation dataset analysis

To evaluate the calibration of the in silico training models, we performed case–control analyses using the validation dataset. In these analyses, OR estimates were fixed according to the population estimates from the training models (Table 1), but the other parameters (intercept and country covariates) were re-estimated, since the case–control proportions might differ between the training and validation datasets. From the validation model, we extracted the predicted probability that each individual was a case and hence derived expected numbers of cases and controls in each risk group. These were used to plot observed versus expected OR estimates and perform a goodness of fit chi-squared test.

**Table 1:** Breast cancer risk association results from logistic regression and mixture models of population training samples

	*N*			Logistic regression model			Mixture model		
Risk group	Variants^a^	Cases	Controls	OR^b^	95% CI^c^	*P*-value	Missense OR (95% CI)^d^	*α*^e^	95% CI^f^
*ATM*				Log-likelihood = − 48,624.97			Log-likelihood = − 48,624.64		
Non-carriers	–	33,351	37,001	1	–	–		0	–
Carriers							2.16 (1.78–2.63)^h^		
Variant outside FAT and PIK domains	714	1259	1443	0.98	(0.91–1.06)	0.67		0.0041	(0.001–0.02)
Variant inside FAT or PIK domain and CADD score quintiles 1–4^ g^	171	317	333	1.10	(0.94–1.29)	0.24		0.055	(0.03–0.12)
Variant inside FAT or PIK domain and CADD score quintile 5^ g^	103	239	162	1.64	(1.33–2.02)	3.1 × 10^−6^		0.54	(0.41–0.68)
*BRCA1*				Log-likelihood = − 48,652.14			Log-likelihood = − 48,652.29		
Non-carriers	–	34,191	37,996	1	–	–		0	–
Carriers							10.61 (7.92–14.21)^h^		
Variant outside RING and BRCT domains	479	811	856	1.01	(0.92–1.12)	0.79		0.0015	(9.4 × 10^−5^–0.025)
Variant inside RING or BRCT domain and low Helix score	79	120	103	1.18	(0.90–1.55)	0.23		1.0 × 10^−11^	NA
Variant inside RING or BRCT domain and high Helix score	23	63	16	4.94	(2.83–8.61)	1.9 × 10^−8^		0.48	(0.19–0.78)
*BRCA2*				Log-likelihood = − 48,641.97			Log-likelihood = − 48,638.78		
Non-carriers	–	33,006	36,517	1	–	–		0	–
Carriers							5.87 (4.75–7.24)^h^		
Variant with low Helix score	1160	2062	2323	0.98	(0.92–1.04)	0.47		5.1 × 10^−5^	(2.4 × 10^−9^–0.52)
Variant with high Helix score	62	114	94	1.28	(0.96–1.70)	0.087		0.11	(0.04–0.25)
*CHEK2*				Log-likelihood = − 48,728.96			Log-likelihood = − 48,728.70		
Non-carriers	–	34,582	38,480	1	–	–		0	–
Carriers							1.75 (1.47–2.08)^i^		
Variant with low Helix score	157	403	363	1.26	(1.08–1.46)	0.0025		0.33	(0.25–0.43)
Variant with high Helix score	121	265	177	1.73	(1.42–2.11)	4.7 × 10^−8^		0.95	(0.86–0.98)
*PALB2*				Log-likelihood = − 48,728.67			Log-likelihood = − 48,729.17		
Non-carriers	–	34,622	38,291	1	–	–		0	–
Carriers	424	618	713	0.95	(0.85–1.06)	0.34	4.87 (3.50–6.77)^h^	1.1 × 10^−4^	(1.6 × 10^−9^–0.88)

^a^ Number of unique missense substitutions in population dataset

^b^ Logistic regression odds ratio estimate for missense variant carriers

^c^ 95% confidence interval for logistic regression OR estimate for missense variant carriers

^d^ Mixture model odds ratio and 95% confidence interval for missense variant carriers

^e^ Alpha: estimated proportion of risk associated missense variants

^f^ 95% confidence interval for alpha

^g^ CADD quintiles 1–4 includes all CADD score values ≤ 3.736542; CADD quintile 5 includes all CADD score values > 3.736542

^h^ Missense variant odds ratio constrained to equal odds ratio for protein truncating variants

^i^ Missense variant odds ratio unconstrained

The mixture models were assessed similarly, with the exception that both the OR parameter and the proportion of risk associated variants, *α*, were fixed. However, an adjustment to *α* was incorporated to allow for the different distribution of cases and controls within the validation set compared to the training set. To do this, the proportions of cases and controls that were carrying a risk associated variant in the training set were estimated separately and *α* in the validation set was then computed as a weighted average of these two estimates. As an alternative approach, the predicted ORs in the validation set were computed using the posterior probabilities (PP) of each variant being risk associated (from the training set) as weights. This analysis was restricted to the subset of individuals carrying variants found in the training set or carrying no variant.

As a final analysis, a single unconstrained logistic regression model comprising all the defined risk groups across the five genes, with non-carriers of any missense variant as the baseline group, was fitted, and the risks in the validation set were evaluated.

The estimated familial relative risk ${\lambda }_{j}$ due to deleterious missenses in each gene $j$ was estimated using the formula ${\lambda }_{j}=\frac{({p}_{j}{r}_{j}^{2}+{q}_{j}{({p}_{j}{r}_{j}+{q}_{j})}^{2}}{{(2{p}_{j}{r}_{j}+1-2{p}_{j})}^{2}}$, where ${p}_{j}$ is the estimated total frequency of deleterious missense variants, ${q}_{j}=1={p}_{j}$ and ${r}_{j}$ is the estimated relative risk conferred by deleterious variants. The total contribution of deleterious missense variants was estimated by assuming that the contribution of variants in the different genes is additive, i.e., ${\lambda }_{mis}=1+\sum ({\lambda }_{j}-1)$. The proportion of the overall familial relative risk due to missense variants was then calculated as $\mathrm{log}\left({\lambda }_{mis}\right)/\mathrm{log}(2)$, that is assuming an overall familial relative risk of 2 and that variant combine multiplicatively with other genetic/familial factors, consistent with previous observations.

## Results

### ATM

**Fig. 1:**
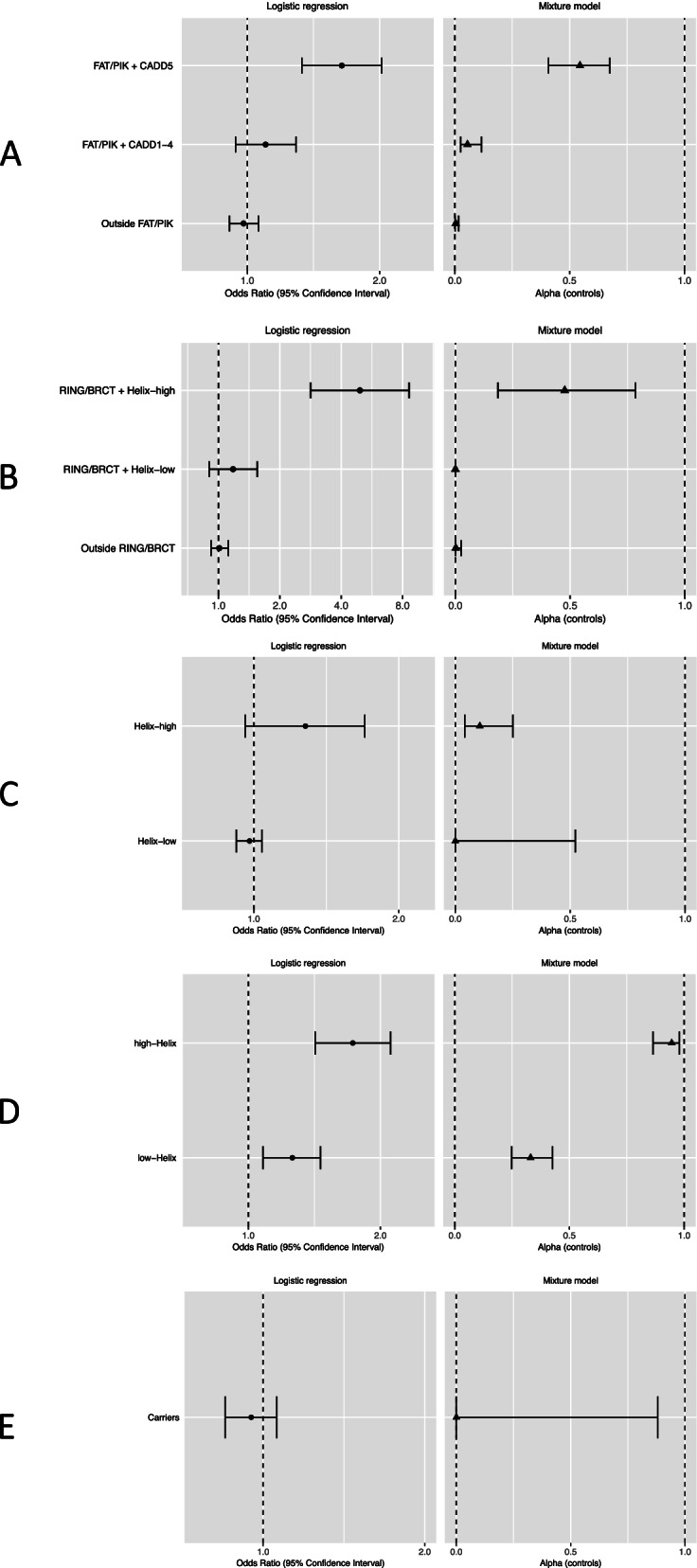
Odds ratios and alpha estimates for each of five genes in population training samples. **A** *ATM*. Odds ratios for breast cancer risk from logistic regression models. Alpha is the estimated proportion of risk associated variants from mixture models, based on variants in control samples. *ATM* risk categories: variants lying within the FAT or PI3K/PI4K protein domains with CADD score in the fifth quintile (FAT/PIK + CADD5); variants lying within the FAT or PI3K/PI4K protein domains with CADD score in any of the first four quintiles (FAT/PIK + CADD1-4); variants lying outside the FAT and PI3K/PI4K protein domains (Outside FAT/PIK). **B*** BRCA1.* Odds ratios for breast cancer risk from logistic regression models. Alpha is the estimated proportion of risk associated variants from mixture models, based on variants in control samples. *BRCA1* risk categories: variants lying within the RING or BRCT domains with a high Helix score (RING/BRCT + Helix-high); variants lying with the RING or BRCT domains with a low Helix score (RING/BRCT + Helix-low); variants lying outside the RING and BRCT domains (Outside RING/BRCT). **C*** BRCA2*. Odds ratios for breast cancer risk from logistic regression models. Alpha is the estimated proportion of risk associated variants from mixture models, based on variants in control samples. *BRCA2* risk categories: variants with a high Helix score (Helix-high); variants with a low Helix score (Helix-low). **D*** CHEK2*. Odds ratios for breast cancer risk from logistic regression models. Alpha is the estimated proportion of risk associated variants from mixture models, based on variants in control samples. *CHEK2* risk categories: variants with a high Helix score (Helix-high); variants with a low Helix score (Helix-low). **E*** PALB2*. Odds ratios for breast cancer risk from logistic regression models. Alpha is the estimated proportion of risk associated variants from mixture models, based on variants in control samples. *PALB2* risk categories: carriers of any missense variant (Carriers)

**Fig. 2:**
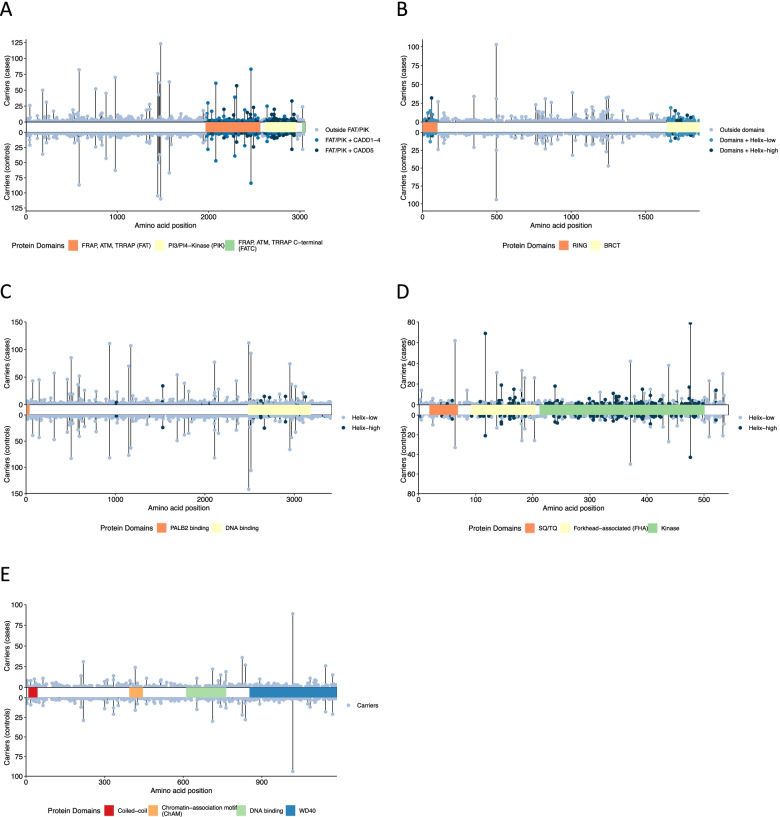
Case and control carriers across all samples for each observed missense variant by gene. **A*** ATM*. *ATM* risk categories: variants lying within the FAT or PI3K/PI4K protein domains with CADD score in fifth quintile (FAT/PIK + CADD5); variants lying within the FAT or PI3K/PI4K protein domains with CADD score in any of first four quintiles (FAT/PIK + CADD1-4); variants lying outside the FAT and PI3K/PI4K protein domains (Outside FAT/PIK). **B*** BRCA1*. *BRCA1* risk categories: variants lying within the RING or BRCT domains with a high Helix score (RING/BRCT + Helix-high); variants lying with the RING or BRCT domains with a low Helix score (RING/BRCT + Helix-low); variants lying outside the RING and BRCT domains (Outside RING/BRCT). **C*** BRCA2*. *BRCA2* risk categories: variants with a high Helix score (Helix-high); variants with a low Helix score (Helix-low). **D*** CHEK2*. *CHEK2* risk categories: variants with a high Helix score (Helix-high); variants with a low Helix score (Helix-low). **E*** PALB2*. *PALB2* risk categories: carriers of any missense variant (Carriers)

The analysis of *ATM* missense variants included 4522 carriers of 1146 unique variants. In the carrier only analysis, BayesDel (*p*_ALL_ = 0.024), CADD (*p*_ALL_ = 0.0022), Helix (*p*_ALL_ = 0.0045), and REVEL (*p*_ALL_ = 0.024) scores were all predictive of risk (see Additional File 2: Table S5). For the most strongly associated score, CADD, the risk appeared to be restricted to the fifth quintile (Q5; CADD > 3.736542; *p* = 0.033 compared with third quintile). Functional protein domain was also predictive, with increased risks associated with the FRAP-ATM-TRRAP (FAT; *p*_ALL_ = 9.5 × 10^−4^) and phosphatidylinositol 3-kinase and 4-kinase (PIK; *p*_ALL_ = 0.0016) domains compared with variants outside a known domain. Including CADD and protein domain, only variants in the category that included CADD Q5 variants in the FAT or PIK domains (FAT/PIK + CADD5) were associated with risk relative to non-carriers (OR 1.64 (1.33–2.02), *p* = 3.1 × 10^−6^; Table 1, Figs. 1a and 2a). In the most parsimonious mixture model, risk associated variants conferred an equivalent risk to PTVs (OR 2.16 (1.78–2.63)); an estimated 54% (95% CI (41–68%)) of variants in the FAT/PIK + CADD5 risk group were risk associated, compared to less than 6% of variants in other risk categories (Table 1, Figs. 1a and 2a). There was no evidence that missense variants were associated with a different risk compared with PTVs (*p* = 0.48). The mixture model was a slightly better fit to the data than the LR model (2 × log-likelihood difference = 0.67). There was no association between age-at-diagnosis and risk category (see Additional File 1: Table S6).

Thirteen *ATM* missense variants were classified as (likely) pathogenic on the ClinVar database (see Additional File 1: Table S7). These variants, in aggregate, were associated with an increased risk (OR 1.85 (0.98–3.50, *p* = 0.060; *p*_ALL_ = 0.00053)). However, the association of (likely) pathogenic variants was not present when the analysis was restricted to the five variants not in the FAT or PIK domains (OR = 0.97 (0.19–5.08)), though the carrier numbers were small and the confidence interval wide. Conversely, variants in the FAT/PIK + CADD5 risk group, in aggregate, remained risk associated, even when variants defined as (likely) pathogenic were excluded (OR 1.60 (1.29–1.99)). Two of the variants classified as (likely) pathogenic were observed in controls only (Additional File 1: Table S7). One of these (c.8546G > C) is located in the PIK domain, the other (c.3848 T > C) is not within any domain; however, both have a Q5 CADD score.

The pathogenic variants listed on ClinVar include c.7271 T > G (p.Val2424Gly), previously reported as associated with high risk of breast cancer [29, 30]. In the training dataset, c.7271 T > G was identified in 12 cases (6 population-based) and 6 controls and was not associated with risk (*p* = 0.37, *p*_ALL_ = 0.081); its population-based OR estimate of 1.63 (0.56–4.73) was lower than previous estimates (for example [31]). Another variant previously reported as risk associated, c.6919C > T (p.Leu2307Phe) [32], was associated with an increased population risk (OR = 3.71 (1.87–7.38), *p* = 0.00018). Both variants are located in the FAT domain and have a CADD score in Q5, but after excluding them from the model, there remained a significantly increased risk for carriers in the FAT/PIK + CADD5 risk group (OR 1.48 (1.18–1.85), *p* = 0.00064).

### BRCA1

The analysis of *BRCA1* missense variants included 2288 carriers of 644 unique variants. For missense variant carriers, all five continuous in silico scores were associated with risk (Align-GVGD *p*_ALL_ = 1.3 × 10^−8^, BayesDel *p*_ALL_ = 0.0013, CADD *p*_ALL_ = 0.011, Helix *p*_ALL_ = 2.1 × 10^−9^, REVEL *p*_ALL_ = 1.5 × 10^−5^). Variants in two protein domains were also significantly associated with risk compared with variants outside these domains (RING finger domain *p*_ALL_ = 3.5 × 10^−4^; BRCA1 C-terminal domains (BRCT I-II) *p*_ALL_ = 0.0030; see Additional File 2: Table S5). The Helix tool categorizes variants with a high score (> 0.5) as “deleterious” and variants with a low score (< 0.5) as “benign”; hereafter, we refer to these categories as Helix-high and Helix-low, respectively. Including Helix category and protein domain, we found that only variants that were inside the RING or BRCT I-II domains and also in the Helix-high category (RING/BRCT + Helix-high) were associated with risk (OR compared with non-carriers 4.94 (2.83–8.61), *p* = 1.9 × 10^−8^; *p*_ALL_ = 2.5 × 10^−9^; Table 1, Figs. 1b and 2b). In a mixture model in which the OR for risk associated missense variants was constrained to that for PTVs (OR 10.61 (7.92–14.21)), the estimated proportions of risk associated variants in the RING/BRCT + Helix-high risk category was 48% (19–78%) and close to 0% for all other variants (Table 1, Figs. 1b and 2b). There was no evidence that the risk associated missense OR differed from the PTV OR (*p* = 0.98). The LR and mixture models were similarly good fits to the data (2 × log-likelihood difference = 0.30).

In a case-only analysis, the OR associated with variants in the RING/BRCT + Helix-high risk category reduced as age increased (per year OR 0.98 (0.96–1.00), *p* = 0.036; see Additional File 1: Table S6).

According to the ENIGMA guidelines and/or ClinVar classifications [13, 14], 13 of the *BRCA1* missense variants in the dataset (four in the RING domain and nine in the BRCT domains) would be classified as (likely) pathogenic (see Additional File 1: Table S7). In total, the 13 variants were carried by 60 cases and 6 controls and were strongly associated with risk in the subset of population samples (OR 16.68 (5.16–53.94), *p* = 2.6 × 10^−6^). In our dataset, the most frequent of these variants was c.181 T > G (p.Cys61Gly), carried by 29 cases and 2 controls (OR 15.06 (3.58–63.36)). After excluding all (likely) pathogenic variants, there also remained an increased risk associated with variants in the RING/BRCT + Helix-high category (OR 2.39 (1.19–4.78), *p* = 0.014)).

RF and NVM predictions from the analysis of Hart et al. were available for 577 unique *BRCA1* missense variants. Variants predicted to be damaging by the RF model (OR 1.82 (1.33–2.49), *p* = 1.9 × 10^−4^) or the NVM model (OR 2.14 (1.52–3.01), *p* = 1.2 × 10^−5^) were associated with increased risk of breast cancer but not as strongly as for variants in the Helix-high category (OR 2.76 (1.93–3.95), *p* = 2.6 × 10^−8^; see Additional File 2: Table S5).

BRCA1 Saturation Genome Editing (SGE) score [22] was available for 100 unique variants and was strongly associated with risk (*p*_ALL_ = 1.5 × 10^−4^; see Additional File 2: Table S5). Carriers of variants with an SGE loss of function (LOF_SGE_) consequence had a higher risk than carriers of variants with a functional (FUNC_SGE_) consequence (OR_ALL_ 10.79 (3.31–35.16)). Carriers of variants with an intermediate function (INT_SGE_) consequence also had, on average, a higher risk than carriers of FUNC_SGE_ variants (OR_ALL_ 3.17 (0.32–31.15)) though the number of INT_SGE_ carriers was small (total *n* = 6). Since the *BRCA1* SGE experiment specifically targeted the domain-coding regions of the gene, only four variants outside of the domains were scored. Thus, all *BRCA1* missense variants were assigned to one of four potential risk levels, with SGE score prioritized where available: INT_SGE_/LOF_SGE_; RING/BRCT + Helix-high (SGE score missing); RING/BRCT + Helix-low (SGE score missing); or FUNC_SGE_ or carriers of variants outside of the domains. Compared with non-carriers, there was increased risk for carriers of variants in the INT_SGE_/LOF_SGE_ category (OR 7.22 (2.48–21.01), *p* = 2.9 × 10^−4^) and in the RING/BRCT + Helix-high category (OR 5.35 (2.48–11.57), *p* = 2.0 × 10^−5^; see Additional File 1: Table S8). In a mixture model in which the OR for risk associated missense variants was constrained to that for PTVs (OR 10.69 (7.97–14.33)), the estimated proportions of risk associated variants in the INT_SGE_ /LOF_SGE_ and the RING/BRCT + Helix-high risk categories were 75% (24–97%) and 51% (6–94%), respectively (Additional File 1: Table S8). The SGE LR model and SGE mixture model were similarly good fits to the data (2 × log-likelihood difference = 0.12) and both were better fits to the data compared to the Helix-only models (LR models 2 × log-likelihood difference = 3.40, mixture models 2 × log-likelihood difference = 3.58).

### BRCA2

The analysis of *BRCA2* missense variants included 5467 carriers of 1425 unique variants. Align-GVGD (*p*_ALL_ = 0.0072), BayesDel (*p*_ALL_ = 0.059), CADD (*p*_ALL_ = 0.036), and Helix (*p*_ALL_ = 0.0016) scores were associated with risk for carriers of *BRCA2* missense variants (see Additional File 2: Table S5). Risks did not differ by protein domain (*p*_ALL_ = 0.91). Compared with non-carriers, carriers of Helix-high variants had a modestly increased risk of breast cancer (OR 1.28 (0.96–1.70), *p* = 0.087; *p*_ALL_ = 0.020) whereas carriers of a Helix-low variant had no increased risk (OR 0.98 (0.92–1.04), *p* = 0.47; *p*_ALL_ = 0.40; Table 1, Figs. 1c and 2c). Under a mixture model in which risk associated missense variants conferred the same risk as PTVs (OR 5.87 (4.75–7.24)), an estimated 11% (4–25%) of the Helix-high variants were associated with risk, compared with < 0.1% of Helix-low variants (Table 1, Figs. 1c and 2c). A model that allowed the OR for missense variants to differ from that of PTVs did not converge. The constrained mixture model was a better fit to the data than the logistic regression model (2 × log-likelihood difference = 6.38). There was no association between age-at-diagnosis and risk category (see Additional File 1: Table S6).

Twelve *BRCA2* variants would be classified as (likely) pathogenic according to ENIGMA guidelines or ClinVar (see Additional File 1: Table S7). In aggregate, the relative risk estimate for these variants was similar to that for PTVs (OR 8.91 (2.61–30.42), *p* = 4.8 × 10^−4^). Ten of these variants were categorized as Helix-high and two as Helix-low. Two of the variants categorized as (likely) pathogenic and Helix-high were observed in controls only (see Additional File 1: Table S7). After excluding the (likely) pathogenic variants from the LR model, there remained no increased risk associated with variants classified as Helix-high (OR 0.60 (0.27–1.34)).

RF and NVM predictions were available for 1338 and 1339 unique *BRCA2* missense variants, respectively. There was no association with risk for variants predicted to be damaging by either the RF model (*p* = 0.16) or the NVM model (*p* = 0.32; see Additional File 2: Table S5).

BRCA2 HDR assay score was available for 82 unique variants and was strongly associated with risk (*p*_ALL_ = 6.7 × 10^−4^; see Additional File 2: Table S5). Carriers of variants with a prediction of likely pathogenic or pathogenic (LP/P) had a higher risk than carriers of variants with a prediction of likely benign or benign (LB/B) (OR_ALL_ 5.57 (2.36–13.17)). Since the BRCA2 HDR experiment specifically targeted the DNA binding domain-coding region of the gene, no variants outside of the domains were scored. Thus, all *BRCA2* missense variants were assigned to one of four potential risk levels, with functional classification prioritized where available: LP/P; Helix-high (no functional classification); Helix-low (no functional classification); or LB/B. Compared with non-carriers, there was increased risk for carriers of variants in the LP/P category only (OR 4.72 (1.88–11.84), *p* = 9.3 × 10^−4^); see Additional File 1: Table S9). In a mixture model in which the OR for risk associated missense variants was constrained to that for PTVs (OR 5.86 (4.75–7.24)), the estimated proportions of risk associated variants in LP/P risk category was 43% (11–82%) (see Additional File 1: Table S9). The functional LR model was a slightly better fit to the data than the mixture model (2 × log-likelihood difference = 1.85) but both were better fits to the data compared to the Helix-only models (LR models 2 × log-likelihood difference = 13.88, mixture models 2 × log-likelihood difference = 5.65).

### CHEK2

The analysis of *CHEK2* missense variants included 1552 carriers of 325 unique variants. In the carrier-only analysis, BayesDel (*p*_ALL_ = 0.0091), CADD (*p*_ALL_ = 0.0073), Helix (*p*_ALL_ = 0.0021), and REVEL (*p*_ALL_ = 0.016) scores were associated with risk (see Additional File 2: Table S5). Compared with non-carriers, carriers of a Helix-high variant had a larger increased risk (OR 1.73 (1.42–2.11), *p* = 4.7 × 10^−8^) than carriers of Helix-low variants, but the latter were also associated with an increased risk (OR 1.26 (1.08–1.46), *p* = 0.0025; see Table 1, Figs. 1d and 2d). There was no significant association with protein domain (*p*_ALL_ = 0.98).

In the mixture model analysis, the constrained model in which risk associated missense variants conferred the same risk as PTVs could be rejected (*p* = 0.027). Under the best fitting model, the OR for missense variants was 1.75 (1.47–2.08), with 95% (86–98%) of Helix-high variants and 33% (25–43%) of Helix-low variants being risk associated (see Table 1, Figs. 1d and 2d). The mixture model was a similar fit to the LR model (2 × log-likelihood difference = 0.52). We also explored mixture models with two levels of risk variant: one with an OR equal to that of PTVs and another conferring a lower risk compared to that of PTVs. The two-level model fitted slightly better in the full training dataset (2 × log-likelihood difference = 1.10) but not in the population-based studies (two-level model converged to the one-level model). The OR associated with Helix-high variants decreased as age increased (per year OR 0.99 (0.98–1.00), *p* = 0.017; see Additional File 1: Table S6).

Two variants, c.470 T > G (p.Ile157Ser) and c.433C > T (p.Arg145Trp), were listed as (likely) pathogenic on ClinVar; both variants have high Helix scores but the number of carriers in our population-based sample was too small to evaluate their association with risk (see Additional File 1: Table S7). One rare variant, c.349A > G (p.Arg117Gly), was previously identified as risk associated in BCAC samples, as part of the OncoArray genome-wide association study (GWAS) project [31]. In the current dataset, this variant, which is in the Helix-high category, had an OR 2.69 (1.46–4.94). After excluding the BCAC GWAS samples from the current dataset, the OR was 3.40 (1.52–7.61). Excluding c.349A > G from the LR model did not change the overall relative risk associated with the Helix-high category (OR 1.64 (1.33–2.02)).

### PALB2

The analysis of *PALB2* missense variants included 1659 carriers of 472 unique variants. We found no overall evidence of risk associated with missense variants in *PALB2* (OR 0.95 (0.85–1.06), *p* = 0.34; *p*_ALL_ = 0.98). In the carrier-only analysis, CADD was the only score associated with risk (*p*_ALL_ = 0.020; see Additional File 2: Table S5); however, there was no significant difference in risk between CADD quintiles (*p*_ALL_ = 0.16). There was no evidence for a difference in risk for carriers of variants inside any protein domain versus those outside (*p*_ALL_ = 0.25). In a mixture model in which the missense variant risk was constrained to that for PTVs (OR 4.87 (3.50–6.77)), the estimated proportion of risk associated variants was 0.011% (95% CI 0–88%; Table 1, Figs. 1e and 2e). The log-likelihoods for the mixture model and logistic regression model were similar (2 × log-likelihood difference = 1.01).

Three (likely) pathogenic variants were listed on ClinVar but none of these were present in our samples. Another variant, c.104 T > C (p.Leu35Pro), has been suggested to be pathogenic based on evidence from one family and tumor genomic analysis [33], but this variant was also not found in our samples.

A subset of the variants from the functional screening studies were available in the training data set: 26 of the 48 assayed by Boonen et al. [24], 34 of the 84 assayed by Wiltshire et al. [26] and 18 of the 44 assayed by Rodrigue et al. [25]. None of the functional assay scores or the authors’ corresponding classifications of pathogenicity were associated with risk in the BRIDGES samples (see Additional File 2: Table S5).

### Frequency analysis

In burden analyses of variants with frequencies up to 5%, variants in *ATM* with frequency < 0.1% were associated, in aggregate, with risk (*p* = 0.0024) but no group of variants of greater frequency was associated (see Additional File 1: Table S10). For *CHEK2*, variants with frequency < 0.1% (*p* = 1.1 × 10^−14^) and those with frequency 0.1–0.5% (*p* = 3.6 × 10^−5^) were associated with risk; there were no variants with frequency 0.5–5%. None of the other genes showed an association between any variant frequency group and risk (see Additional File 1: Table S10).

When analyses were restricted to frequencies up to 0.5%, there was no association between risk and frequency, either on a continuous scale or as the difference in risk between the two frequency groups < 0.1% and 0.1–0.5%, for *BRCA2*, *CHEK2*, or *PALB2* (see Additional File 1: Table S11). For *ATM*, we found frequency inversely associated with risk (continuous *p*_ALL_ = 0.0098) and a higher risk for variants with frequency < 0.1% compared with variants of frequency 0.1–0.5% (*p*_ALL_ = 0.031). After adjusting for the CADD and domain risk groups, the associations remained statistically significant (*p*_ALL_ = 0.0097 and *p*_ALL_ = 0.012, respectively). For *BRCA1*, we found frequency inversely associated with risk (continuous *p*_ALL_ = 0.022) and a significantly higher risk for variants with frequency < 0.1% compared with variants of frequency 0.1–0.5% (*p*_ALL_ = 0.0066). However, after adjusting for the Helix and domain risk groups, neither of these associations remained statistically significant (*p*_ALL_ = 0.36 and *p*_ALL_ = 0.39, respectively).

We evaluated the risks for individual missense variants with frequency between 0.1 and 5% (see Additional File 1: Table S12). In *BRCA1*, one variant, c.2521C > T (p.Arg841Trp), was associated with a decreased risk of breast cancer (OR 0.67 (0.52–0.87), *p* = 0.0027). Two previously-reported variants in *CHEK2* were identified: c.470 T > C (p.Ile157Thr) and c.538C > T (p.Arg180Cys) [34]. c.470 T > C was associated with an OR of 1.24 (1.09–1.42), consistent with the estimate for the Helix-low risk category, while c.538C > T was associated with a higher OR 1.44 (1.12–1.84). No *ATM*, *BRCA2*, or *PALB2* missense variants were individually associated with increased risk.

### Model validation

We evaluated the calibration of the best fitting models from the training set, for each gene, in the validation set: these included the LR models, the mixture model using the estimated proportions (*α*) from the training set, and the mixture model using the posterior probabilities derived from the training set. For each gene and each model, carriers of variants in the predicted risk groups were associated with an increased risk, and there were no differences between the observed and predicted ORs (see Additional File 1: Table S13 and Figs. S2-S6). In silico scores, likelihood ratios and posterior probabilities for every variant included in the population training dataset are given in Additional File 2: Tables S14-18.

**Fig. 3:**
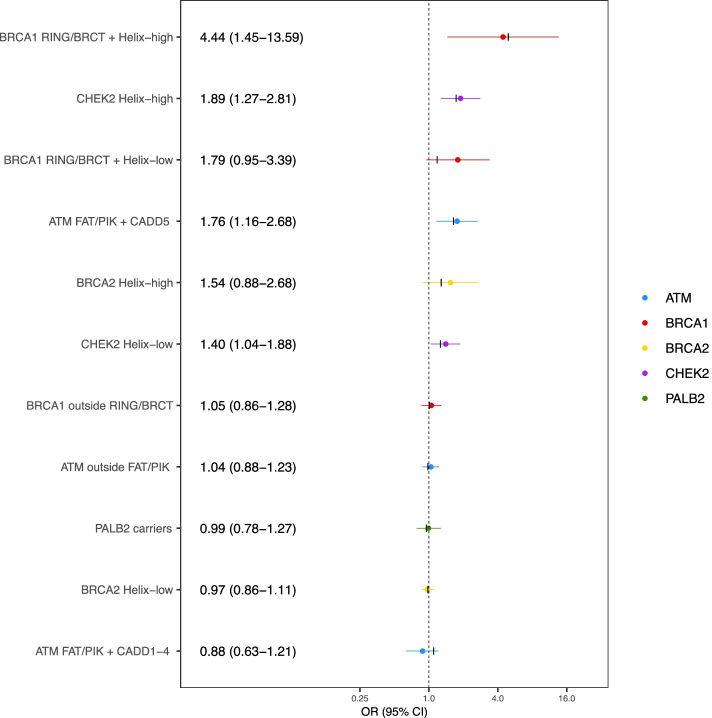
Breast cancer risk estimates from composite gene model in validation samples. Black marks indicate corresponding ORs from training models. Risk categories: ATM FAT/PIK + CADD5: *ATM* variants lying within the FAT or PI3K/PI4K protein domains with CADD score in fifth quintile; ATM FAT/PIK + CADD1-4: *ATM* variants lying within the FAT or PI3K/PI4K protein domains with CADD score in any of first four quintiles; ATM outside FAT/PIK: variants lying outside the FAT and PI3K/PI4K protein domains; BRCA1 RING/BRCT + Helix-high: *BRCA1* variants lying within the RING or BRCT domains with a high Helix score; BRCA1 RING/BRCT + Helix-low: *BRCA1* variants lying with the RING or BRCT domains with a low Helix score; BRCA1 outside RING/BRCT: *BRCA1* variants lying outside the RING and BRCT domains; BRCA2 Helix-high: *BRCA2* variants with a high Helix score; BRCA2 Helix-low: *BRCA2* variants with a low Helix score; CHEK2 Helix-high: *CHEK2* variants with a high Helix score; CHEK2 Helix-low: *CHEK2* variants with a low Helix score; PALB2 carriers: carriers of any missense variant in *PALB2*

Using a composite five gene model, we estimated ORs for eleven risk categories (Fig. 3). In total, 184 samples carried a missense variant in more than one of the five genes and were excluded from this analysis. Four categories were significantly associated with an increased risk relative to non-carriers, consistent with the estimates derived from the training set: *ATM* FAT/PIK + CADD5 (OR 1.76 (1.16–2.68), *p* = 0.0078), *CHEK2* Helix-low (OR 1.40 (1.04–1.88), *p* = 0.025), *CHEK2* Helix-high (OR = 1.89 (1.27–2.81), *p* = 0.0017), and *BRCA1* within domain and Helix-high (OR 4.44 (1.45–13.59), *p* = 0.0089) risk groups. The OR estimate for *BRCA2* Helix-high variant carriers was higher than that in the training dataset, but the confidence interval was considerably wider (OR 1.54 (0.88–2.68)). As predicted, variants in the remaining categories were not associated with risk.

## Discussion

To date, the risks associated with missense variants in breast cancer predisposition genes have been largely unclear. In this study of over 112,000 women, we were able to use a range of in silico scores produced by statistical algorithms and knowledge of functional protein domains to determine the risks associated with subsets of rare missense variants. We identified groups of missense variants conferring increased risks of breast cancer in *ATM*, *BRCA1*, *BRCA2*, and *CHEK2*, but not in *PALB2*. The ORs for *BRCA1* and *CHEK2* decreased with age at diagnosis, consistent with previous observations for PTVs [7]. Previous analysis of the full BRIDGES dataset showed that protein domains in *ATM* and *BRCA1* were predictive of risk [7]; the analysis presented here showed that in silico scores improved these predictions, in a formal model evaluation that allowed the models to be tested in an independent validation set. Under the best fitting mixture models, for *ATM*, *BRCA1*, and *BRCA2*, a small proportion of rare missense variants were associated with risks comparable to those for PTVs. In contrast, for *CHEK2*, a high proportion of *CHEK2* missense variants were risk associated and the estimated risk was markedly lower than that associated with PTVs. In *PALB2*, the evidence for association was weak; the mixture model analysis indicated that the proportion of missense variants associated with a high risk is likely to be very small. However, we cannot rule out the possibility that some variants are risk-associated since the power for detecting an association with risk for *PALB2* is lower than, for example, *BRCA1* and *BRCA2*. One variant in *BRCA1* (p.Arg841Trp) was individually associated with a reduced risk of breast cancer (0.67 (0.52–0.87)). Given that this finding is inconsistent with all the other associations and that the variant is not in any of the key functional domains, it seems quite likely that this is a chance association; further replication in other datasets will be required to confirm or refute the association.

We used five in silico scores to predict the pathogenicity of individual variants. Helix, BayesDel, and CADD were all predictive for the four genes for which we were able to identify subsets of risk-associated variants; Helix was most predictive for *BRCA1*, *BRCA2*, and *CHEK2* while CADD outperformed all the other scores for *ATM*. In addition to the in silico scores, we also tested the BRCA1 SGE functional assay score*.* We found that the SGE score slightly improved the performance of the model for predicting risk for *BRCA1* missense variant carriers, compared with the Helix-only model. Consistent with this, we observed two variants that were classified as loss-of-function variants by SGE but appeared in our low-risk group; these were present in three cases and no controls. Conversely, another four variants that were classified as normal function by SGE but appeared in our high-risk group were present in eight cases and five controls. Overall, variants categorized by SGE as disruptive to function, or lying within a protein domain and scored high by Helix, were strongly associated with increased risk. Under the mixture model, the proportions of risk-associated variants were also high, although the confidence intervals for the proportion of associated variants were wide. It is notable that 11 of the 31 variants in these categories have previously been identified as (likely) pathogenic by ClinVar and/or ENIGMA.

Similarly, for *BRCA2*, we also tested the HDR functional assay score and found it improved the performance of the model for predicting risk for *BRCA2* variant carriers, compared to the Helix-only model. Consistent with this, four variants in the Helix-high category were classified as benign by the functional study and observed in 22 cases and 35 controls. Conversely, one variant in the Helix-low category was classified as pathogenic by the functional study and observed in two cases and no controls. After accounting for variants predicted to be pathogenic by the functional assay, there remained no significant increase in risk for carriers of variants in the Helix-high variant category, compared to non-carriers, although the OR of 1.37 for the Helix-high category was higher than the ORs of 0.97 and 0.96 for the Helix-low and predicted benign categories, respectively. We note that the variants tested using the HDR assay were subsequently classified using a combination of the assay result and American College of Medical Genetics and Genomics/Association for Molecular Pathology (ACMG/AMP) guidelines; ACMG guidelines also used by the ENIGMA *BRCA1/2* expert panel and in evidence for pathogenicity in ClinVar. Consequently, there is considerable overlap between classifications; nine of the 14 variants classified as (likely) pathogenic by the functional study have been previously identified as (likely) pathogenic by ClinVar and/or ENIGMA.

The BRCA2 HDR functional assay included only variants lying in the DNA binding domain of *BRCA2*. The majority of high-Helix variants were also in the DNA binding domain (37/62) and fewer [21] in the “coldspot” regions of exons 10 and 11 as described by Dines et al. [35] (by definition, none of the *BRCA1* variants in the high-risk category fall in the corresponding exon 11 coldspot).

In *ATM*, the risk conferred by missense variants was confined to specific protein-coding domains, namely the FAT and PIK domains, consistent with previous studies [5] and as shown previously in BRIDGES [7]. Variants within these domains could be further distinguished using the CADD score; variants in the top quintile were associated with risk whereas variants in the first four quintiles were not. In a mixture model, 54% of variants in the top CADD quintile were estimated to be associated with risk. One variant in this group, c.7271 T > G (p.Val2424Gly), has been previously reported as a breast cancer risk variant but the OR estimate for this variant, 1.63 (0.56–4.73), was markedly lower than previously estimated (relative risks ranging from 8.0 to 12.7) [29–31]. The reasons for this difference are unclear but might be due, in part, to previous studies oversampling for cases with a family history of breast cancer.

The results for *CHEK2* were in marked contrast to those for *BRCA1*, *BRCA2*, and *ATM*. In the best fitting mixture model, the proportion of associated variants was high, and the estimated risk was clearly lower than for PTVs. A model in which there were two levels of risk, with the higher level equal to the PTV risk, fitted slightly better in the full training dataset but not in the population-based training studies. In addition, however, three individual *CHEK2* variants were associated with differing levels of risk: c.470 T > C (p.Ile157Thr) OR 1.24 (1.09–1.42); c.538C > T (p.Arg180Cys) OR 1.44 (1.12–1.84); and c.349A > G (p.Arg117Gly) OR 2.69 (1.46–4.94). The c.470 T > C variant was too common to be included in the main analyses, possibly explaining why the heterogeneity in risk was not readily detectable by the mixture models; however, the confidence interval for c.470 T > C from the individual-level analysis did not include the LR and mixture model OR estimates of 1.73 and 1.75, respectively, for the risk-associated variants. Taken together, these observations suggest that there is substantial variation in risk associated with *CHEK2* missense variants.

The relative performances of the in silico prediction algorithms are perhaps less marked than might appear; for example, Helix, which was the most predictive algorithm for three of the genes, was also predictive for *ATM*. Some of the differences in the associations may be due to chance. Align-GVGD was initially developed for *BRCA1*/*2* so it is perhaps not surprising that the algorithm does relatively well for *BRCA1* but less well for *CHEK2*, for example. Helix was not developed for a specific gene so may be a more useful tool in general.

We controlled for the potential effects of population stratification by stratifying analyses by country and by excluding individuals with the minority ancestry for that country. Thus, European studies excluded individuals of non-European ancestry and Asian studies excluded individuals of non-Asian ancestry. In addition, for the studies in Malaysia and Singapore, we further stratified into the three ethnic groups (Chinese, Malay, Indian). In previous analysis of PTVs, we found no differences in effect sizes when additionally correcting for ancestry informative principal components, suggesting that this correction was adequate, particularly since most of the associations were based on many variants [7]. Nevertheless, it remains possible that some estimates may be biased due to residual population stratification [36, 37].

Under the best fitting mixture model, approximately 7% of all rare missense variants in *ATM* were associated with similar risk to that of PTVs. The estimated carrier frequency of pathogenic missense variants in *ATM* was 0.0030, or approximately 89% of the PTV frequency. The corresponding proportion of associated rare missense variants for *BRCA1* and *BRCA2* was 2% and 0.6%, with an estimated carrier frequency of 0.00026 (~ 18%) and 0.00028 (~ 9%), respectively. Thus, missense variants add modestly to the contribution of *BRCA1* and *BRCA2* variants to breast cancer incidence, but make a relatively more substantial contribution for *ATM*. The differences between genes in the relative contributions of missense variants to risk presumably reflect the relative proportion of residues within functional domains in which disrupted function is associated with cancer risk, and the size of those domains. For *CHEK2*, approximately 60% of rare missense variants were risk associated and the estimated carrier frequency of pathogenic missense variants in *CHEK2* was comparable to the frequency of PTVs. The predicted proportion of breast cancer cases possessing pathogenic germline missense variants in these genes is approximately 0.6%, 0.3%, 0.2%, and 1.3% for *ATM*, *BRCA1*, *BRCA2*, and *CHEK2*, respectively. The estimated additional contribution to the familial relative risk of breast cancer made by pathogenic missense variants in these five genes is approximately 2.7%.

The task of identifying which specific individual missense variants are risk associated is a complex one and is difficult to resolve fully even with a large dataset, since most variants are rare and there are many possible models to consider. Despite the size of our study, it was difficult to distinguish, for any gene, between the LR models (in which all variants in a given category confer a given risk) and the mixture models (in which all risk-associated variants confer the same risk, but the proportion that are associated varies by category). This difficulty arises because the number of carriers for individual variants is small, and as a result, the estimated risk of pathogenic missense variants and probability of pathogenicity (*α*) are strongly confounded. Further, selecting the best models and estimating the risks based on these models is likely to result in overfitting and biased risk estimates. In order to strengthen the validity of our findings, we used a training-validation study design. We were able to replicate the predicted OR estimates in the validation dataset, suggesting that any bias due to overfitting was small. Nevertheless, the validation dataset was relatively small, so further validation of the best models reported here in large independent datasets is critical.

Ultimately, high-throughput functional assays that can evaluate all possible missense substitutions may provide more precise definitions of risk categories. The analyses of the BRCA1 SGE scores and the BRCA2 HDR assay scores suggest that this approach should be useful, although the scores for *BRCA1* were highly concordant with the best in silico score in this case. The available *PALB2* functional assays did not predict risk, but this may just reflect the low power of these analyses when the proportion of risk-associated variants is very low. As further prediction algorithms based on in silico and/or in vitro data are developed, large population-based epidemiological datasets such as BRIDGES can be used to validate their predictions. However, further large studies are likely to be required to provide more precise variant-specific risk estimates.

## Conclusions

This study confirms that subsets of missense variants in established breast cancer susceptibility genes are associated with increased risks of the disease and provides estimates of relative risks for those subsets, as well as probabilities for association with risk at the variant level. The pattern of risk varies substantially by gene. Accurately and precisely defining these risks is critical to the counselling and management of women in whom these variants are identified.

## Supplementary Information

**Additional file 1.**

**Additional file 2.**

## Data Availability

The datasets generated during and/or analyzed during the current study are not publicly available due to constraints by the ethics committees of individual studies. The datasets are available via the BCAC Data Access Co-ordinating Committee (bcac@medschl.cam.ac.uk), upon reasonable request. Summary-level genotype data are available via http://bcac.ccge.medschl.cam.ac.uk and in Additional File 2: Tables S14-18. Individual-level data are available via the BCAC Data Access Co-ordinating Committee (bcac@medschl.cam.ac.uk).

## Abbreviations

PTVs: Protein truncating variants
VUS: Variants of uncertain significance
BCAC: Breast Cancer Association Consortium
VEP: Ensembl Variant Effect Predictor
CADD: Combined Annotation-Dependent Depletion
REVEL: Rare Exome Variant Ensembl Learner
SGE: Saturation Genome Editing
LR: Logistic regression
OR: Odds ratio
EM: Expectation-maximization
PP: Posterior probability
Q5: Fifth quintile
LOF_SGE_: Loss of function
FUNC_SGE_: Functional
INT_SGE_: Intermediate function
GWAS: Genome-wide association study
