# Supplementary material for: Breast cancer risks associated with missense variants in breast cancer susceptibility genes
Source: Genome Med. 2022 May 18;14:51. doi: 10.1186/s13073-022-01052-8 (PMC9116026; doi:10.1186/s13073-022-01052-8)
Supplement: Supplementary file 1 — Additional file 1. [file 13073_2022_1052_MOESM1_ESM.docx]

**Fig. S1:** Flow diagram of statistical analysis. Training analyses in blue shaded area and validation analyses in yellow shaded area. Boxes outlined in orange show analyses based on population samples only.

BRIDGES samples sequenced and passed genotype and phenotype quality control measures

N = 112,804

Validation sample (20%)

N = 21,850

Population samples only

Mixture model with fixed beta coefficient and variant posterior probabilities (PPs)

Comparison of expected and observed numbers of cases and controls by PP intervals

Mixture model with fixed beta coefficient and alphas

Comparison of expected and observed numbers of cases and controls by risk category

Logistic regression with fixed beta coefficient

Comparison of expected and observed numbers of cases and controls by risk category

Training sample (80%)

N = 87,136

Population samples = 74,277

Logistic regression (LR) to select in silico features and evaluate protein domains

Assign risk categories according to categories of most predictive feature(s)

Posterior probability of risk association for every variant

Mixture modelling for proportions of risk-associated variants (alpha) by risk category

Logistic regression for odds ratios and 95% CI by risk category

3,818 protein truncating variant (PTV) carriers excluded

3,818 PTV carriers re-included

**Fig. S2:** Plot of expected versus observed ORs for carriers of *ATM* missense variants in the test dataset, by risk category under the logistic regression model with fixed beta; by risk category under the mixture model with fixed alphas and beta; by intervals of variant posterior probabilities (PPs) under the mixture model with fixed PPs.

**Fig.S3:** Plot of expected versus observed ORs for carriers of *BRCA1* missense variants in the test dataset, by risk category under the logistic regression model with fixed beta; by risk category under the mixture model with fixed alphas and beta; by intervals of variant posterior probabilities (PPs) under the mixture model with fixed PPs.

**Fig. S4:** Plot of expected versus observed ORs for carriers of *BRCA2* missense variants in the test dataset, by risk category under the logistic regression model with fixed beta; by risk category under the mixture model with fixed alphas and beta; by intervals of variant posterior probabilities (PPs) under the mixture model with fixed PPs.

**Fig. S5:** Plot of expected versus observed ORs for carriers of *CHEK2* missense variants in the test dataset, by risk category under the logistic regression model with fixed beta; by risk category under the mixture model with fixed alphas and beta; by intervals of variant posterior probabilities (PPs) under the mixture model with fixed PPs.

**Fig. S6:** Plot of expected versus observed ORs for carriers of *PALB2* missense variants in the test dataset, by risk category under the logistic regression model with fixed beta; by risk category under the mixture model with fixed alphas and beta; by intervals of variant posterior probabilities (PPs) under the mixture model with fixed PPs.

# Table S1. Description of studies included in the analyses.

| **Study** | **Abbreviation** | **Country** | **Study design** | **Case definition** | **Control definition** | **Selected familial cases** | **Design category** | **References** |
| --- | --- | --- | --- | --- | --- | --- | --- | --- |
| Amsterdam Breast Cancer Study | ABCS | Netherlands | Hospital-based consecutive cases; population-based controls (for iCOGS/OncoArray/BRIDGES from blood bank). | iCOGS/OncoArray/BRIDGES:  Breast cancer patients diagnosed before age 50 in 1995-2011 at the Netherlands Cancer Institute - Antoni van Leeuwenhoek hospital (NKI-AVL). | iCOGS/OncoArray/BRIDGES: Population-based cohort of women recruited through the Sanquin blood bank, all ages. | No | Mixed | (1, 2) |
| Amsterdam Breast Cancer Study - Familial | ABCS-F | Netherlands | Clinical Genetic Center-based cases | iCOGS/OncoArray/BRIDGES: All non-BRCA1/2 breast cancer cases from the family cancer clinic of the NKI-AVL tested in the period 1995-2009; all ages and diagnosed with breast cancer in 1972-2010. | No controls. [Use controls of ABCS] | Yes | Case-only; clinical genetic center-based | (3) |
| Asia Cancer Program | ACP | Thailand | Hospital-based case-control study | Cases recruited 1999-2000 and 2008-present at The National Cancer Institute (Central region), The Prince Songkla University Research Centre (South region), The HRH Princess Maha Chakri Sirindhorn Medical Centre (MSMC)-Srinakarinviroj University (Eastern region), Khon-Kaen University Cancer Centre (North-eastern region). 1. Women who underwent biopsy and have been pathologically diagnosed as having breast cancer. 2. Aged less than 71 years of age. | Controls recruited 1999-2000 and 2008-present at The National Cancer Institute (Central region), The Prince Songkla University Research Centre (South region), The HRH Princess Maha Chakri Sirindhorn Medical Centre (MSMC)-Srinakarinviroj University (Eastern region), Khon-Kaen University Cancer Centre (North-eastern region). 1. Women aged less than 71 years of age without cancer history of any kinds 2. Women who attend the out-patient clinic under the minor injuries such as cuts, broken bones. 3. Women who are institutionalised at the hospital with diseases not related to cancer or metabolic syndromes such as diabetes, heart diseases or conditions related to gynaecology and are well enough to give information to researchers. | No | Mixed | None |
| Bavarian Breast Cancer Cases and Controls | BBCC | Germany | Hospital-based cases; population based controls | Consecutive, unselected cases with invasive breast cancer recruited at the University Breast Centre, Franconia in Northern Bavaria during 1999-2013. | Healthy women with no diagnosis of cancer aged 55 or older. Invited by a newspaper advertisement in Northern Bavaria, and recruited during 1999-2013. | No | Mixed | (4, 5) |
| Breast Cancer in Galway Genetic Study | BIGGS | Ireland | Hospital-based cases; population based controls | Unselected cases recruited from West of Ireland since 2001. Cases were recruited from University College Hospital Galway and surrounding hospitals | Women > 60 years with no personal history of any cancer and no family History of breast or ovarian cancer were identified from retirement groups in the West of Ireland (same catchment area as cases) during the period 2001-2008. | No | Mixed | (6-8) |
| Breast Oncology Galicia Network | BREOGAN | Spain | Population-based case-control | A population-based study conducted since 1997 in two cities in Galicia, Spain (Vigo and Santiago) covering approximately 700,000 inhabitants. The study currently includes over 1600 incident breast cancer cases diagnosed from 1997-2014 in two Galician hospitals with blood, tumor tissue and risk factor questionnaire. | Controls were frequency-matched to cases according to 5-year age group, inclusion in the universal Galician Public Health Service (SERGAS) registry database, and place of residence. They were healthy, unrelated female individuals from the same base population as cases randomly selected from SERGAS´ primary healthcare centers in the health areas of Santiago and Vigo. Recruitment began in 1997. | No | Population-based | (8-12) |
| Breast Cancer Study of the University of Heidelberg | BSUCH | Germany | Hospital-based cases;healthy blood donator controls | Cases diagnosed with breast cancer/breast cancer metastasis in 2008-2011 at the University Women`s Clinic Heidelberg. | Healthy, unrelated, ethnically matched female blood donors recruited in 2007, 2009 & 2012 by German Red Cross Blood Service of Baden-Württemberg-Hessen, Institute of Transfusion Medicine & Immunology, Mannheim. | No | Mixed | (13) |
| Crete Cancer Genetics Program | CCGP | Greece | Hospital-based case-control study | Incident breast cancer cases treated between 2004 and 2013 at the University Hospital of Heraklion on Crete; all enrolled within 6 months of diagnosis. | Healthy, unrelated, ethnically matched female blood donors recruited in 2014 by the laboratory of Hemostasis at the General Hospital of Heraklion "Venizelio". | No | Mixed | Unpublished |
| CECILE Breast Cancer Study | CECILE | France | Population-based case-control study | All incident cases of breast cancer diagnosed in 2005-2007 among women <75 years of age and residing in Ille-et-Vilaine or Côte d'Or. Cases were recruited from the main cancer treatment center (Centre Eugène-Marquis in Rennes and Centre Georges-François-Leclerc in Dijon) and from private or public hospitals in each area. | General population control women residing in the same geographic areas frequency-matched to the cases by 5-year age groups. Controls were recruited in 2005-2007 by phone using a random digit dialing procedure and predefined numbers by socioeconomic status to control for possible selection bias. | No | Population-based | (14) |
| Copenhagen General Population Study | CGPS | Denmark | Population-based case-control study | Consecutive, incident cases from 1 hospital with centralized care for a population of 400,000 women from 2001 to the present. | Community controls residing in the same region as cases and with no history of breast cancer were identified from the Copenhagen General Population Study recruited 2003-2007. All controls were known to still be breast cancer-free at the end of 2007. | No | Mixed | (15) |
| Spanish National Cancer Centre Breast Cancer Study | CNIO-BCS | Spain | Case-control study | Two groups of cases:1) 574 consecutive breast cancer patients, unselected for family history, from 3 public hospitals, 2 in Madrid and one in Oviedo, from 2000 to 2005. 2) 291 cases with at least one first degree relative also affected with breast cancer, recruited through the CNIO family cancer clinic in Madrid from 2000 to 2004. | Women attending the Menopause Research Centre between 2000 and 2004 and female members of the College of Lawyers attending a free, targeted medical check-up in 2005, all free of breast cancer and all in Madrid | Subset (N=291) | Mixed | (16) |
| Colombian Breast Cancer Case-Control Study | COLBCCC | Colombia | Case-control study | 1,022 unselected women diagnosed with breast cancer after January 1, 2004; enrolled between 2007 and 2012. | 1,023 healthy women attending the country-wide National Pap-Smear Screening Program in Colombia; enrolled between 2007 and 2012. Controls were matched to cases by +/- 2 years. Controls were women participating in the Colombian National Pap-Smear Screening Program (participation rate in 2005 was 77%) | No | Mixed | Unpublished |
| Family History Risk Study | FHRISK | UK | Clinic-based cohort study with a nested case-control study | Women diagnosed with breast cancer and attending the Family History Clinic in Manchester for increased risk of breast cancer. Recruitment period 2009-2012. | Women attending the same Family History Clinic as the cases but without a breast cancer diagnosis. Recruitment period is the same as for the cases. | Yes | Cohort and case-control | (17, 18) |
| German Consortium for Hereditary Breast & Ovarian Cancer | GC-HBOC | Germany | Clinic-based case study and prospective cohort study | Women diagnosed with breast cancer in one of the GC-HBOC centres (Cologne, Munich, Kiel, Heidelberg, Düsseldorf, Ulm, Würzburg, Münster and Hannover). Recruitment period 1996-present. | Healthy, unrelated, ethnically and age-matched female control individuals (LIFE study, Leipzig, Germany). | Yes | Mixed | (19-22) |
| Gene Environment Interaction and Breast Cancer in Germany | GENICA | Germany | Population-based case-control study | Incident breast cancer cases enrolled between 2000 and 2004 from the Greater Bonn area (by of the hospitals within the study region); all enrolled within 6 months of diagnosis. | Selected from population registries from 31 communities in the greater Bonn area; matched to cases in 5-year age classes between 2001 and 2004. | No | Population-based | (23, 24) |
| Generation Scotland | GENSCOT | Scotland | Prospective family-based cohort study; nested case-control | Incident and prevalent cases of histologically-confirmed breast cancer at the time of latest updated cancer registry linkage (currently 2013). Recruitment though the General Practitioners in the areas of Glasgow, Tayside, Ayrshire, Arran and Northeast Scotland. | Two groups of controls: (1) 2:1 unrelated individuals matched to cases on age in five-years at baseline and recruitment centre; (2) first-degree female relatives with no breast cancer diagnosis at the time of selection. | No | Prospective cohort | (25) |
| Genetic Epidemiology Study of Breast Cancer by Age 50 | GESBC | Germany | Population-based study of women <50 years | All incident cases diagnosed <50 years of age in 1992-5 in two regions: Rhein-Neckar-Odenwald and Freiburg, by surveying the 38 clinics serving these regions | Selected from random lists of residents of the study regions supplied by population registries; two controls were selected for each case, matched by age and study region. Recruitment was carried out 1992-1998. | No | Population-based | (26) |
| Hannover Breast Cancer Study | HABCS | Germany | Hospital-based case-control study | Cases who received radiotherapy for breast cancer at Hannover Medical School between 1996-2003 (HaBCS I), or were diagnosed with breast cancer at a certified Breast Cancer Clinics in the Hannover region between 2012-2016 (HaBCS II), unselected for age or family history. | Anonymous female blood bank donors at Hannover Medical School, collected from 8/2005-12/2005, with known age and ethnic background. | No | Mixed | (27) |
| Helsinki Breast Cancer Study | HEBCS | Finland | Hospital-based case-control study, plus additional familial cases | (1) Consecutive cases (883) from the Department of Oncology, Helsinki University Central Hospital 1997-8 and 2000, (2) Consecutive cases (986) from the Department of Surgery, Helsinki University Central Hospital 2001 – 2004, (3) Familial breast cancer patients (536) from the Helsinki University Central Hospital, Departments of Oncology and Clinical Genetics (1995-) | Healthy females from the same geographical region in Southern Finland in 2003. | Subset (N=609) | Mixed | (28-30) |
| Hereditair Borst-en eierstokkanker Onderzoek Nederland | HEBON | Netherlands | Clinical genetic center-based recruitment of familial breast or ovarian cancer patients (cases) | Breast (or sometimes ovarian) cancer patients who were tested for mutations in BRCA1 and BRCA2 in one of the clinical genetic centers in the Netherlands between 1996 and 2016. All counselees received an invitation to participate in the HEBON study. | No controls. [Use of controls (bloodbank donors) from ORIGO, ABCS or RBCS]. | Yes (All participants are familial cases) | Case-only; clinical genetic center-based | Unpublished |
| Hannover-Minsk Breast Cancer Study | HMBCS | Belarus | Hospital-based cases; population based controls | Ascertainment at the Byelorussian Institute for Oncology and Medical Radiology Aleksandrov N.N. in Minsk or at one of 5 regional oncology centers in Gomel, Mogilev, Grodno, Brest or Vitebsk through the years 2002-2008. | Controls from the same population aged 18-72 years. Healthy (without personally history of cancer) female probunds recruited from the same geographical regions as cases during the years 2002-2008. About 75% of controls were women invited for general medical examination at five regional gynecology clinics (in Gomel, Mogilev, Grodno, Brest or Vitebsk) and cancer-free volunteers ascertained at the Institute for Inherited Diseases in Minsk; 20% were cancer-free female blood bank donors recruited at Republic Blood Bank, Minsk, Belarus; finally 5% of controls were healthy cancer-free relatives of some breast cancer patients. | No | Mixed | (31) |
| Hannover-Ufa Breast Cancer Study | HUBCS | Russia | Hospital-based cases; population based controls | Consecutive Russian breast cancer patients aged 24-86 years ascertained at one of the two participating oncological centers in Bashkorstostan and Siberia through the years 2000-2008. | Population controls aged 18-84 years recruited from a population study of different populations of Russia. Healthy volunteers (without any malignancy) were selected from the same geographical regions during the years 2002-2008. | No | Mixed | (31) |
| Karolinska Breast Cancer Study | KARBAC | Sweden | Population and hospital-based cases; geographically matched controls | 1. Familial cases from Department of Clinical Genetics, Karolinska University Hospital, Stockholm. 2. Consecutive cases from Department of Oncology, Huddinge & Söder Hospital, Stockholm 1998-2000 | Blood donors of mixed gender from same geographical region. Excess material was received from all blood donors over a 3 month period in 2004 (approximately 3000) and DNA was extracted from a random sample of 1500 | Subset (N=568) | Mixed | (32, 33) |
| Karolinska Mammography Project for Risk Prediction of Breast Cancer - Cohort Study | KARMA | Sweden | Cohort study | Inclusion of 70,877 women Oct 2010 - March 2013. 3000 women had BC at cohort entry. In all, 800 women have been diagnosed with breast cancer since study entry (Oct 2015). Approximately 250 women are diagnosed with BC annually | Non - BC cases in the Karma Cohort | no | Prospective cohort | Submitted |
| Kuopio Breast Cancer Project | KBCP | Finland | Population-based prospective clinical cohort | 1. Women seen at Kuopio University Hospital between 1990 and 1995 because of breast lump, mammographic abnormality, or other breast symptom who were found to have breast cancer. 2. Consecutive malignant breast cancer cases diagnosed at KUH from 2011 onwards. | Age and long-term area-of-residence matched controls selected from the National Population Register and interviewed in parallel with the cases | No | Population-based | (34, 35) |
| Kathleen Cuningham Foundation Consortium for research into Familial Breast Cancer/Australian Ovarian Cancer Study | kConFab/AOCS | Australia and New Zealand | Clinic-based recruitment of familial breast cancer patients (cases); population-based case-control study of ovarian cancer (controls only) | Cases were from multiple-case breast and breast-ovarian families recruited though family cancer clinics from across Australia and New Zealand from 1998 to the present. Cases were selected for inclusion in BCAC studies if (i) family was negative for mutations in BRCA1 and BRCA2 (ii) case was the index for the family, defined as youngest breast cancer affected family member. | Female controls were ascertained by the Australian Ovarian Cancer Study identified from the electoral rolls from all over Australia from 2002-2006. | Yes | Mixed | (36, 37) |
| Korean Hereditary Breast Cancer Study | KOHBRA | Korea | Population-based case-control study | Breast cancer patients at high risk were recruited from nationwide University Hospitals from May 2007 to May 2012. High-risk status mean 1) familial breast cancer, 2) early onset breast cancer (age <40), 3) breast and past/current ovarian cancer patients 4) cases with past/current double primary cancers, 5) bilateral breast cancer, 6) male breast cancer cases. All cases participated in the BCAC project were BRCA non-carriers and male breast cancers were not included. | Health examinee controls from communities were enrolled and individual matched to the cases on specific age. A part of the controls were recruited from unaffected family members of BRCA mutation carriers. | Subset (N=1192) | Mixed | (38) |
| Mammary Carcinoma Risk Factor Investigation | MARIE | Germany | Population-based case-control study | Incident cases diagnosed from 2001-2005 in the study region Hamburg in Northern Germany, and from 2002-2005 in the study region Rhein-Neckar-Karlsruhe in Southern Germany. | 2 controls per case were randomly drawn from population registries and frequency matched by birth year and study region to the case. Controls were recruited from 2002 to 2006. | No | Population-based | (39) |
| Cyprus Breast Cancer Case Control Study | MASTOS | Cyprus | Population-based case-control study | Women between 40-70 years of age who had a histologically confirmed diagnosis of primary breast cancer between January 1999 and December of 2005. The majority of cases were ascertained from the Bank of Cyprus Oncology Centre, which operates as a referral centre and offers treatment and follow-up for up to 90% of all breast cancer cases diagnosed in Cyprus. The rest of the patients, were recruited at the Oncology Departments of the Nicosia, Limassol, Larnaca and Paphos district hospitals. | Cypriot women from the general population, who were invited to participate in the National programme for breast cancer screening with the use of mammography and received a negative result. Volunteers were enrolled in the study during the same calendar period as the cases, from the 5-district mammography screening centers that operate in Cyprus. | No | Population-based | (40) |
| Milan Breast Cancer Study Group | MBCSG | Italy | Clinic-based recruitment of familial/early onset breast cancer patients (cases); population-based controls | Familial and/or early onset breast cancer patients (aged 22-87) negative for mutations in BRCA genes, ascertained in two large cancer centres in Milan from 2000 to date. | Healthy blood donors aged 18-71 years, recruited at two blood centres in Milan from 2004 (centre 1) and 2007 (centre 2) to date | Yes (ca. 90%) | Mixed | (41, 42) |
| Melbourne Collaborative Cohort Study | MCCS | Australia | Prospective cohort study: nested case-control study | Incident cases diagnosed between baseline (1990-1994) and last follow-up (2012) among the 24469 women participating in the cohort. | For each case a control was randomly selected from women from the cohort who did not develop breast cancer before the age at diagnosis of the case and matched the case on year of birth and country of birth. | No | Prospective cohort | (43) |
| Malaysian Breast Cancer Genetic Study | MYBRCA | Malaysia | Hospital-based case-control study | Breast cancer cases identified at the Breast Cancer Clinic in University Malaya Medical Centre Jan 2003-July 2014 and Subang Jaya Medical Centre Sep 2012-Sept 2014; cases are a mixture of prevalent and incident cases. Includes hospital-based and familial series. | Controls are cancer-free individuals (37-74 years) selected from women attending mammographic screening at the same hospitals. | Yes (subset) | Mixed | (44, 45) |
| Norwegian Breast Cancer Study | NBCS | Norway | Hospital-based case-control study | Incidence cases from three different hospitals: 1) Cases (114) mean age 64 (28-92) at Ullevål Univ. Hospital 1990-94, 2) cases (182) mean age 59 (26-75) referred to Norwegian Radium Hospital 1975-1986, 3) cases (124), mean age 56 (29-82) with stage I or II disease, in the Oslo micro-metastases study at Norwegian Radium Hospital between 1995-1998, 4) Breast cancer cases referred to the Norwegian hospitals Akershus University Hospital in Lørenskog, Ullevaal university hospital in Oslo and Rikshospitalet-Radiumhospitalet in Oslo from 2007-2010. Mean age is 63 years. Consecutive series. 5) Breast cancer cases referred to the Norwegian Radium Hospital hospitalet 2010-2013. Neoadjuvantly treated with Avastin (Bevacizumab). 6) Consecutive series of Breast cancer incidents referred to Akershus university hospital 2004-2014. | Control subjects were healthy women, age 55-71, residing in Tromsø (440), and Bergen (109) attending the Norwegian Breast Cancer Screening Program. Healthy tissue from mammoplastic reduction surgery at a private clinic in Oslo. | No | Mixed | (46-49) |
| Ontario Familial Breast Cancer Registry | OFBCR | Canada | Population-based familial case-control study | Cases diagnosed between 1 Jan 1996-31 Dec 1998 were identified from the Ontario Cancer Registry which registers >97% of all cases residing in the province at the time of diagnosis. All women with invasive breast cancer aged 20–54 years who met the OFBCR definition for high genetic risk (family history of specific cancers particularly breast and ovarian, early onset disease, Ashkenazi ethnicity or a diagnosis of multiple breast cancer) were asked to participate by completing risk factor questionnaires and providing a blood sample. A 25% random sample of individuals in this age category who did not meet the OFBCR definition, 35% of those aged 55–69 at high risk and 8.75% aged 55–69 at low risk were also asked to participate. Individuals diagnosed in 2001 and 2002 were also included if they met high-risk criteria. | Unrelated, unaffected population controls were recruited by the Ontario Familial Breast and Colon Cancer Registries by calling randomly selected residential telephone numbers throughout the same geographical region. Eligible controls were women with no history of breast cancer and were frequency-matched by 5-year age group to the expected age distribution of cases. | Subset (N=628) | Mixed | (50) |
| Leiden University Medical Centre Breast Cancer Study | ORIGO | Netherlands | Hospital-based prospective cohort study | Consecutive cases diagnosed 1996-2006 in 2 hospitals of South-West Netherlands (Leiden & Rotterdam). No selection for family history; Rotterdam cases selected for diagnosis aged <70. Cases with in situ carcinomas eligible. | Three groups of controls: (1) Blood bank healthy donors from Southwest Netherlands recruited in 1996, 2000 or 2007; (2) People who married a person who was part of a family with high breast cancer risk (BRCA1/2/x). From the Southwest of the Netherlands, recruited 1990-1996; (3) Females tested at the local clinical genetics department for familial diseases, excluding familial cancer syndromes (no mutation found in gene(s) related to the disease being tested), recruited 1995-2007. | No | Mixed | (51, 52) |
| NCI Polish Breast Cancer Study | PBCS | Poland | Population-based case-control study | Incident cases from 2000-2003 identified through a rapid identification system in participating hospitals covering ~ 90% of all eligible cases, and cancer registries in Warsaw and Łódź covering 100% of all eligible cases. | Randomly selected from population lists of all residents of Poland, stratified and frequency matched to cases by case city and age in 5 year categories. Recruited 2000-2003. | No | Population-based | (53) |
| The Prostate,Lung,Colorectal and Ovarian (PLCO) Cancer Screening Trial | PLCO | USA | Prospective cohort study: nested case-control | Incident cases arising in the sub-cohort of 78,232 women who gave a blood specimen in 1993-2001 are included if they were diagnosed with breast cancer. Recruitment via multiple screening centers across the US. | Controls were women in this sub-cohort who were not diagnosed with breast cancer. Controls were matched to cases on age at randomization (4 categories) and fiscal year of randomization (2 categories). | No | Prospective cohort | (54) |
| Predicting the Risk Of Cancer At Screening Study | PROCAS | UK | Population based study | Women diagnosed with breast cancer since joining the study of women attending the Breast Screening Programme (NHSBSP) in Greater Manchester. Recruitment period Oct 2009-May 2014. | Women attending routine NHS breast screening in Greater Manchester without a breast cancer diagnosis. Recruited during the same period as for the cases. | No | Population-based | (17) |
| Rotterdam Breast Cancer Study | RBCS | Netherlands | Hospital-based case-control study, Rotterdam area | Familial breast cancer patients selected from the Clinical Genetics Center at Erasmus MC Cancer Institute; recruited 1994 - 2005 (RBCS1) and 1995 - 2009 (RBCS2; for OncoArray). | Spouses or mutation-negative siblings of heterozygous Cystic Fibrosis mutation carriers selected from the Clinical Genetics Center at Erasmus MC Cancer Institute; recruited 1996 - 2006 (RBCS1) and 2005 - 2009 (RBCS2). | Yes | Mixed | (55) |
| Singapore and Sweden Breast Cancer Study | SASBAC | Sweden | Population-based case-control study | Incident cases from October 1993 to March 1995 identified via the 6 regional cancer registries in Sweden, to which reporting is mandatory. | Controls were randomly selected from the total population registry in 5-year age groups to match the expected age-frequency distribution among cases. Patients and controls were recruited from Oct 1993 through April 1995. | No | Population-based | (56) |
| Study of Epidemiology and Risk factors in Cancer Heredity | SEARCH | UK | Population-based case-control study | 2 groups of cases identified through East Anglian Cancer Registry; 1) prevalent cases diagnosed 1991-1996 under 55 years of age at diagnosis, recruited 1996-2002; 2) incident cases diagnosed since 1996 under 70 years of age at diagnosis, recruited 1996-present. | Two groups of controls: (1) selected from the EPIC-Norfolk cohort study of 25,000 individuals age 45-74 recruited between 1992 and1994, based in the same geographic region as cases; (2) selected from GP practices from March 2003 to present, frequency matched to cases by age and geographic region | No | Mixed | (57) |
| Singapore Breast Cancer Cohort | SGBCC | Singapore | Hospital-based breast cancer cohort and population-based controls | Living breast cancer patients diagnosed with primary in situ or invasive breast cancer at 7 restructured hospitals in Singapore between 1980-2016. Cases are a mixture of prevalent and incident cases. | All community-dwelling individuals who are Singaporeans or Singaporean Permanent Residents, 21 years and older. Participants were recruited between 2006 and 2010 through word-of-mouth and personal recommendations. In some cases, recruiters also sought participants through "cold-calling" or through door-to-door invitations. Exclusion criteria were a medical history of cancer, acute myocardial infarction or stroke, or major psychiatric morbidity including schizophrenia, psychotic depression, and advanced Alzheimer's Disease. | No | Hospital-based | No refs. |
| Städtisches Klinikum Karlsruhe Deutsches Krebsforschungszentrum Study | SKKDKFZS | Germany | Hospital-based breast cancer cohort | Women diagnosed with primary *in situ* or invasive breast cancer at the Städtisches Klinikum Karlsruhe from March 1993 to July 2005. | No controls. | No | Patient cohort | (58) |
| IHCC-Szczecin Breast Cancer Study | SZBCS | Poland | Hospital-based case-control study | Prospectively ascertained cases of invasive breast cancer patients diagnosed at the Regional Oncology Hospital (Szczecin) in the years 2002, 2003, 2006 and 2007 or the University Hospital from 2002 to 2007 in Szczecin, West-Pomerania, Poland. Patients with pure intraductal or intralobular cancer were excluded (DCIS or LCIS) but patients with DCIS with micro-invasion were included. | Unaffected, matched to cases for year of birth, sex and region; from families with negative cancer family history; controls were part of a population-based study of the 1.3 million inhabitants of West Pomerania performed in 2003 and 2004 designed to identify familial aggregations of cancer by our centre | No | Mixed | (59-62) |
| Utah Breast Cancer Study | UBCS | USA | Mixed. (1) Pedigrees including multiple sampled breast cancer cases within 2 generations, also may include sampled, unaffected relatives; (2) hospital-based cases (from Huntsman Cancer Institute [HCI] or Intermountain Healthcare [IH]), and breast reduction controls; and (3) Population-based cases (from the Utah Cancer Registry [UCR]) and controls (from the Utah Drivers License Registry [UDLR]) | Cases recruited from late 1970s to present (on-going). Ascertainment from: (1) UCR-confirmed breast cancer cases in high-risk pedigrees; (2) invasive breast cancer cases treated or surgery performed at HCI or IH clinics; (3) prevalent, population-based UCR-confirmed breast cancer cases. | Controls also recruited from late 1970s to present (on-going) from: (1) relatives in high-risk pedigrees; (2) hospital-based cancer-free women undergoing breast reductions; (3) Population-based controls selected from the UDLR to frequency match cases by sex and birth cohort. | Some | Mixed | (63, 64) |

# Table S2. Numbers of cases and controls, and age distributions, by study, after QC.

| **Study** | **Country** | **Cases sequenced in BRIDGES** | **Controls sequenced in BRIDGES** | **Cases in BRIDGES after QC** | **Controls in BRIDGES after QC** | **Case age at diagnosis** | | **Control age at interview** | |
| --- | --- | --- | --- | --- | --- | --- | --- | --- | --- |
|  |  |  |  |  |  | **Mean** | **Range** | **Mean** | **Range** |
| ABCS | Netherlands | 1075 | 1824 | 1007 | 1660 | 42.1 | 18-49 | 47.1 | 18-69 |
| ABCS-F | Netherlands | 313 | 0 | 208 | 0 | 45.2 | 22-86 | - | - |
| ACP | Thailand | 960 | 829 | 933 | 789 | 48.4 | 19-78 | 41.6 | 15-73 |
| BBCC | Germany | 357 | 234 | 244 | 159 | 61.2 | 27-90 | 57.5 | 22-84 |
| BIGGS | Ireland | 384 | 384 | 369 | 366 | 56.3 | 27-87 | 66.7 | 46-91 |
| BREOGAN | Spain | 973 | 570 | 598 | 398 | 56.0 | 30-88 | 55.1 | 30-86 |
| BSUCH | Germany | 263 | 697 | 241 | 549 | 56.8 | 32-88 | 57.8 | 30-69 |
| CCGP | Greece | 697 | 294 | 475 | 275 | 55.8 | 26-85 | 61.4 | 17-94 |
| CECILE | France | 988 | 979 | 941 | 943 | 54.3 | 25-74 | 54.6 | 25-74 |
| CGPS | Denmark | 3735 | 5202 | 3387 | 5076 | 61.5 | 26-98 | 56.3 | 20-94 |
| CNIO-BCS | Spain | 856 | 647 | 687 | 569 | 54.3 | 28-88 | 50.0 | 24-73 |
| COLBCCC | Colombia | 517 | 731 | 484 | 621 | 49.5 | 23-83 | 50.0 | 24-73 |
| FHRISK | UK | 311 | 1028 | 276 | 923 | 49.6 | 29-78 | 40.5 | 19-73 |
| GC-HBOC | Germany | 2742 | 1597 | 2566 | 1561 | 45.2 | 17-87 | 61.8 | 47-79 |
| GENICA | Germany | 1009 | 1005 | 848 | 894 | 58.2 | 23-80 | 58.4 | 24-80 |
| GENSCOT | Scotland | 478 | 1345 | 427 | 766 | 54.7 | 28-89 | 58.4 | 20-93 |
| GESBC | Germany | 635 | 1090 | 552 | 982 | 42.5 | 24-51 | 42.7 | 24-52 |
| HABCS | Germany | 1078 | 900 | 971 | 838 | 58.1 | 23-91 | 33.2 | 17-68 |
| HEBCS | Finland | 2154 | 1254 | 1905 | 1090 | 56.7 | 23-95 | 40.9 | 18-66 |
| HEBON | Netherlands | 2107 | 0 | 1953 | 0 | 47.2 | 22-91 | - | - |
| HMBCS | Belarus | 387 | 381 | 334 | 268 | 47.4 | 17-80 | 46.6 | 20-87 |
| HUBCS | Russia | 404 | 363 | 239 | 192 | 52.4 | 25-82 | 45.1 | 16-78 |
| KARBAC | Sweden | 421 | 539 | 376 | 471 | 59.1 | 27-88 | - | - |
| KARMA | Sweden | 3665 | 6221 | 3329 | 5633 | 55.5 | 23-94 | 60.2 | 29-82 |
| KBCP | Finland | 579 | 75 | 560 | 70 | 58.7 | 23-92 | 51.0 | 30-75 |
| kConFab/AOCS | Australia and New Zealand | 1787 | 8 | 1463 | 7 | 52.9 | 20-94 | 51.9 | 41-77 |
| KOHBRA | Korea | 2019 | 2010 | 1956 | 1835 | 40.6 | 19-83 | 47.8 | 19-87 |
| MARIE | Germany | 2526 | 1981 | 2300 | 1768 | 62.1 | 49-75 | 61.8 | 49-75 |
| MASTOS | Cyprus | 1127 | 1177 | 990 | 1094 | 51.5 | 26-74 | 55.7 | 28-71 |
| MBCSG | Italy | 982 | 776 | 935 | 735 | 42.7 | 18-80 | 44.1 | 18-71 |
| MCCS | Australia | 1185 | 1139 | 1042 | 1029 | 63.6 | 31-88 | 63.3 | 39-88 |
| MYBRCA | Malaysia | 1168 | 1212 | 1076 | 1093 | 51.8 | 24-83 | 56.0 | 38-77 |
| NBCS | Norway | 623 | 614 | 565 | 600 | 60.3 | 24-96 | 61.4 | 55-71 |
| OFBCR | Canada | 562 | 494 | 505 | 416 | 58.8 | 24-83 | 55.1 | 25-81 |
| ORIGO | Netherlands | 0 | 960 | 0 | 919 | - | - | - | - |
| PBCS | Poland | 1899 | 1941 | 1757 | 1849 | 55.9 | 28-75- | 55.6 | 24-75 |
| PLCO | USA | 2322 | 2574 | 2060 | 2221 | 68.4 | 55-87 | 62.3 | 54-74 |
| PROCAS | UK | 656 | 1653 | 518 | 1434 | 58.6 | 29-76 | 59.4 | 46-73 |
| RBCS | Netherlands | 1314 | 975 | 1043 | 899 | 44.4 | 22-99 | - | - |
| SASBAC | Sweden | 1152 | 1344 | 1131 | 1321 | 63.1 | 50-75 | 63.3 | 49-76 |
| SEARCH | UK | 13835 | 7251 | 12817 | 6486 | 54.5 | 23-87 | 53.3 | 16-87 |
| SGBCC | Singapore | 4588 | 4383 | 4271 | 4165 | 53.3 | 18-91 | 50.1 | 21-75 |
| SKKDKFZS | Germany | 1229 | 0 | 966 | 0 | 60.6 | 23-93 |  |  |
| SZBCS | Poland | 372 | 204 | 357 | 191 | 59.2 | 26-91 | 56.7 | 25-85 |
| UBCS | USA | 1006 | 337 | 804 | 306 | 56.3 | 28-92 | 57.0 | 18-94 |

**Table S4:** Functional protein domain definitions.

| Gene | Domain | Amino acids | Reference |
| --- | --- | --- | --- |
| *ATM* | FRAP, ATM, TRRAP (FAT) | 1966-2566 | Lau, W.C. et al, 2016. Structure of the human dimeric ATM kinase. *Cell Cycle*, *15*(8), pp.1117-1124. |
|  | PI3/PI4-Kinase (PIK) | 2614-2960 |  |
|  | FRAP, ATM, TRRAP C-terminal (FAT-C) | 3025-3056 |  |
| *BRCA1* | RING finger | 1-101 | ENIGMA: Evidence-based Network for the Interpretation of Germline Mutant Alleles, available at https://enigmaconsortium.org^a^ |
|  | BRCA1 C-terminal (BRCT) I-II | 1642-1863 |  |
| *BRCA2* | PALB2 binding | 10-40 | ENIGMA: Evidence-based Network for the Interpretation of Germline Mutant Alleles, available at https://enigmaconsortium.org^a^ |
|  | DNA binding | 2481-3186 |  |
| *CHEK2* | SQ/TQ | 19-69 | Cai, Z. et al, 2009. Structure and activation mechanism of the CHK2 DNA damage checkpoint kinase. *Molecular cell*, *35*(6), pp.818-829. |
|  | Forkhead-associated (FHA) | 92-205 |  |
|  | Kinase | 212-501 |  |
| *PALB2* | Coiled-coil | 9-44 | Rodrigue, A. et al, 2019. A global functional analysis of missense mutations reveals two major hotspots in the PALB2 tumor suppressor. *Nucleic acids research*, *47*(20), pp.10662-10677.  Boonen, R.A. et al, 2019. Functional analysis of genetic variants in the high-risk breast cancer susceptibility gene PALB2. *Nature communications*, *10*(1), pp.1-15. |
|  | Chromatin-association motif (ChAM) | 394-446 |  |
|  | DNA binding | 611-764 |  |
|  | WD40 | 853-1186 |  |

^a^drawn from ENIGMA ClinGen External Expert Panel Rules Version 2.5.1, describing conserved domains/motifs known to harbour clinically important missense alterations

**Table S6:** Case-only analyses of age at diagnosis by variant risk category.

|  | Risk of age for carriers of variants in high-risk category | | |
| --- | --- | --- | --- |
| Gene | Per-year OR | 95% CI | P-value |
| *ATM* | 0.99 | 0.98-1.84 | 0.31 |
| *BRCA1* | 0.98 | 0.96-1.08 | 0.036 |
| *BRCA2* | 0.99 | 0.98-2.04 | 0.36 |
| *CHEK2* | 0.99 | 0.98-1.04 | 0.017 |
| *PALB2* | 1.01 | 1.00-1.09 | 0.041 |

**Table S7:** BRIDGES missense variants classified as (Likely) Pathogenic on ClinVar (*ATM*, *CHEK2*, *PALB2, BRCA1, BRCA2*) or by ENIGMA expert guidelines (*BRCA1*, *BRCA2*).

| Gene | Variant | N cases^a^ | N controls^a^ |
| --- | --- | --- | --- |
| *ATM* | c.875C>T (p.Pro292Leu) | 1 | 0 |
| *ATM* | c.2849T>G (p.Leu950Arg) | 1 | 0 |
| *ATM* | c.3848T>C (p.Leu1283Pro) | 0 | 1 |
| *ATM* | c.6200C>A (p.Ala2067Asp) | 1 | 0 |
| *ATM* | c.6679C>T (p.Arg2227Cys) | 5 | 0 |
| *ATM* | c.7271T>G (p.Val2424Gly) | 12 | 6 |
| *ATM* | c.7570G>C (p.Ala2524Pro) | 4 | 0 |
| *ATM* | c.8122G>A (p.Asp2708Asn) | 2 | 0 |
| *ATM* | c.8147T>C (p.Val2716Ala) | 9 | 5 |
| *ATM* | c.8494C>T (p.Arg2832Cys) | 8 | 2 |
| *ATM* | c.8546G>C (p.Arg2849Pro) | 0 | 1 |
| *ATM* | c.9022C>T (p.Arg3008Cys) | 4 | 1 |
| *ATM* | c.9023G>A (p.Arg3008His) | 3 | 1 |
| *Estimate for aggregated variants* | | *OR (95% CI)* | *P-value* |
| *All samples* | | *2.74 (1.55-4.86)* | *0.00053* |
| *Population samples* | | *1.85 (0.98-3.50)* | *0.060* |
| *BRCA1* | c.5339T>C (p.Leu1780Pro) | 9 | 2 |
| *BRCA1* | c.5216A>G (p.Asp1739Gly) | 1 | 0 |
| *BRCA1* | c.5213G>A (p.Gly1738Glu) | 3 | 0 |
| *BRCA1* | c.5207T>C (p.Val1736Ala) | 1 | 0 |
| *BRCA1* | c.5095C>T (p.Arg1699Trp) | 2 | 0 |
| *BRCA1* | c.5089T>C (p.Cys1697Arg) | 2 | 0 |
| *BRCA1* | c.5072C>T (p.Thr1691Ile) | 2 | 0 |
| *BRCA1* | c.5057A>G (p.His1686Arg) | 4 | 0 |
| *BRCA1* | c.4964C>T (p.Ser1655Phe) | 1 | 0 |
| *BRCA1* | c.181T>G (p.Cys61Gly) | 28 | 3 |
| *BRCA1* | c.181T>C (p.Cys61Arg) | 1 | 0 |
| *BRCA1* | c.130T>A (p.Cys44Ser) | 2 | 1 |
| *BRCA1* | c.53T>C (p.Met18Thr) | 4 | 0 |
|  | *Estimate for aggregated variants* | *OR (95% CI)* | *P-value* |
| *All samples* | | 9.97 (4.29-23.17) | 9.2x10^-8^ |
| *Population samples* | | 16.68 (5.16-53.94) | 2.6x10^-6^ |
| *BRCA2* | c.7529T>C (p.Leu2510Pro) | 2 | 1 |
| *BRCA2* | c.7879A>T (p.Ile2627Phe) | 0 | 1 |
| *BRCA2* | c.7940T>C (p.Leu2647Pro) | 1 | 0 |
| *BRCA2* | c.7958T>C (p.Leu2653Pro) | 0 | 1 |
| *BRCA2* | c.8023A>G (p.Ile2675Val) | 3 | 0 |
| *BRCA2* | c.8057T>C (p.Leu2686Pro) | 1 | 0 |
| *BRCA2* | c.8167G>C (p.Asp2723His) | 10 | 0 |
| *BRCA2* | c.8243G>A (p.Gly2748Asp) | 3 | 0 |
| *BRCA2* | c.9004G>A (p.Glu3002Lys) | 4 | 0 |
| *BRCA2* | c.9154C>T (p.Arg3052Trp) | 5 | 0 |
| *BRCA2* | c.9226G>A (p.Gly3076Arg) | 3 | 0 |
| *BRCA2* | c.9371A>T (p.Asn3124Ile) | 11 | 1 |
|  | *Estimate for aggregated variants* | *OR (95% CI)* | *P-value* |
| *All samples* | | 10.31 (3.65-29.11) | 1.1x10^-5^ |
| *Population samples* | | 8.91 (2.61-30.42) | 4.8x10^-4^ |
| *CHEK2* | c.470T>G (p.Ile157Ser) | 1 | 0 |
| *CHEK2* | c.433C>T (p.Arg145Trp) | 17 | 7 |

^a^ Number of carriers in complete training dataset

**Table S8:** Breast cancer risk association results from *BRCA1* risk model including SGE score of population samples in the training dataset.

|  | N | | | Logistic regression model | | | Mixture model | | |
| --- | --- | --- | --- | --- | --- | --- | --- | --- | --- |
| Risk group | Variants^a^ | Cases | Controls | OR^b^ | 95% CI^c^ | P-value | Missense OR (95% CI)^d^ | α^e^ | 95% CI^f^ |
| *BRCA1 – SGE model* |  |  |  | Log-likelihood = -48650.44 | | | Log-likelihood = -48650.50 | | |
| Non-carriers | - | 34191 | 37996 | 1 | - | - |  | 0 | - |
| Carriers |  |  |  |  |  |  | 10.69 (7.97-14.33)^g^ |  |  |
| FUNC_SGE_ or variant outside RING and BRCT domains | 538 | 878 | 922 | 1.02 | (0.93-1.12) | 0.68 |  | 9.4x10^-4^ | (3.2x10^-5^-0.027) |
| Variant inside RING or BRCT domain and low Helix score | 20 | 55 | 41 | 1.29 | (0.85-1.96) | 0.22 |  | 2.9x10^-25^ | NA |
| Variant inside RING or BRCT domain and high Helix score | 3 | 37 | 8 | 5.35 | (2.48-11.57) | 2.0x10^-5^ |  | 0.51 | (0.062-0.94) |
| SGE_INT_ or SGE_LOF_ | 20 | 24 | 4 | 7.22 | (2.48-21.01) | 2.9x10^-4^ |  | 0.75 | (0.24-0.97) |

^a^ Number of unique missense substitutions in population dataset

^b^ Logistic regression odds ratio estimate for missense variant carriers

^c^ 95% confidence interval for logistic regression OR estimate for missense variant carriers

^d^ Mixture model odds ratio and 95% confidence interval for missense variant carriers

^e^ Alpha: estimated proportion of risk associated missense variants

^f^ 95% confidence interval for alpha

^g^ Missense variant odds ratio constrained to equal odds ratio for protein truncating variants

**Table S9:** Breast cancer risk association results from *BRCA2* risk model including functional-based predictions of population samples in the training dataset.

|  | N | | | Logistic regression model | | | Mixture model | | |
| --- | --- | --- | --- | --- | --- | --- | --- | --- | --- |
| Risk group | Variants^a^ | Cases | Controls | OR^b^ | 95% CI^c^ | P-value | Missense OR (95% CI)^d^ | α^e^ | 95% CI^f^ |
| *BRCA2 – functional model* |  |  |  | Log-likelihood = -48635.03 | | | Log-likelihood = --48635.95 | | |
| Non-carriers | - | 33006 | 36517 | 1 | - | - |  | 0 | - |
| Carriers |  |  |  |  |  |  | 5.86 (4.75-7.24)^g^ |  |  |
| Likely benign/benign | 58 | 378 | 463 | 0.96 | (0.83-1.10) | 0.53 |  | 8.4x10^-15^ | NA |
| Low Helix score | 1105 | 1705 | 1894 | 0.97 | (0.91-1.05) | 0.47 |  | 5.0x10^-5^ | (1.6x10^-9^-0.61) |
| High Helix score | 47 | 68 | 54 | 1.36 | (0.94-1.96) | 0.11 |  | 0.039 | (0.010-0.21) |
| Likely pathogenic/pathogenic | 12 | 25 | 6 | 4.72 | (1.88-11.84) | 9.3x10^-4^ |  | 0.43 | (0.11-0.82) |

^a^ Number of unique missense substitutions in population dataset

^b^ Logistic regression odds ratio estimate for missense variant carriers

^c^ 95% confidence interval for logistic regression OR estimate for missense variant carriers

^d^ Mixture model odds ratio and 95% confidence interval for missense variant carriers

^e^ Alpha: estimated proportion of risk associated missense variants

^f^ 95% confidence interval for alpha

^g^ Missense variant odds ratio constrained to equal odds ratio for protein truncating variants

**Table S10:** Case-control burden analyses of variants with frequency up to 5%.

^a^ Risk relative to non-carriers

|  | Frequency <0.01% | | | Frequency 0.01% - 0.05% | | | Frequency 0.05% - 0.1% | | | 0.01-0.05 | | |
| --- | --- | --- | --- | --- | --- | --- | --- | --- | --- | --- | --- | --- |
| Gene | OR^a^ | 95% CI | P-value | OR^a^ | 95% CI | P-value | OR^a^ | 95% CI | P-value | OR^a^ | 95% CI | P-value |
| *ATM* | 1.11 | (1.04-1.18) | 0.0024 | 0.99 | (0.91-1.09) | 0.82 | 1.04 | (0.96-1.14) | 0.33 | 0.97 | (0.90-1.04) | 0.36 |
| *BRCA1* | 1.06 | (0.97-1.16) | 0.17 | 0.96 | (0.85-1.09) | 0.57 | 0.98 | (0.85-1.14) | 0.82 | 1.01 | (0.95-1.07) | 0.86 |
| *BRCA2* | 1.01 | (0.95-1.08) | 0.69 | 1.04 | (0.96-1.13) | 0.30 | 1.03 | (0.93-1.14) | 0.61 | 0.96 | (0.89-1.03) | 0.24 |
| *CHEK2* | 1.52 | (1.37-1.69) | 1.10E-14 | 1.27 | (1.13-1.42) | 3.60E-05 | NA | NA | NA | NA | NA | NA |
| *PALB2* | 0.98 | (0.89-1.09) | 0.74 | 1.04 | (0.90-1.21) | 0.58 | 1.00 | (0.88-1.14) | 0.97 | 0.98 | (0.91-1.04) | 0.48 |

**Table S11:** P-values for association of variant frequency up to 0.5% with breast cancer risk in all missense variant carriers in training dataset.

| Gene | *P*-value | | |
| --- | --- | --- | --- |
|  | Continuous frequency | Log-scale | Frequency <0.1% versus frequency 0.1%-0.5% |
| *ATM* | 0.0098 | 0.15 | 0.031 |
| *BRCA1* | 0.022 | 0.029 | 0.0066 |
| *BRCA2* | 0.069 | 0.73 | 0.18 |
| *CHEK2* | 0.090 | 0.43 | 0.21 |
| *PALB2* | 0.62 | 0.53 | 0.69 |

**Table S12:** Breast cancer risk association results for individual missense variants with frequency between 0.1% and 5% based on population samples in the training dataset.

| Gene | Variant | Case carriers | Control carriers | OR | 95% CI | *P*-value | P_ALL_^a^ |
| --- | --- | --- | --- | --- | --- | --- | --- |
| *ATM* | c.146C>G (p.Ser49Cys) | 672 | 795 | 1.00 | (0.89-1.11) | 0.93 | 0.33 |
| *ATM* | c.378T>A (p.Asp126Glu) | 46 | 32 | 1.56 | (0.98-2.47) | 0.060 | 0.067 |
| *ATM* | c.998C>T (p.Ser333Phe) | 89 | 94 | 1.11 | (0.82-1.50) | 0.49 | 0.30 |
| *ATM* | c.1229T>C (p.Val410Ala) | 118 | 153 | 0.88 | (0.68-1.13) | 0.31 | 0.32 |
| *ATM* | c.1810C>T (p.Pro604Ser) | 54 | 58 | 1.00 | (0.68-1.48) | 0.99 | 0.34 |
| *ATM* | c.2119T>C (p.Ser707Pro) | 613 | 660 | 1.05 | (0.94-1.18) | 0.39 | 0.38 |
| *ATM* | c.2572T>C (p.Phe858Leu) | 826 | 1039 | 0.93 | (0.85-1.03) | 0.16 | 0.12 |
| *ATM* | c.3161C>G (p.Pro1054Arg) | 1387 | 1655 | 0.97 | (0.90-1.05) | 0.42 | 0.20 |
| *ATM* | c.3925G>A (p.Ala1309Thr) | 70 | 110 | 0.73 | (0.53-1.00) | 0.050 | 0.041 |
| *ATM* | c.4138C>T (p.His1380Tyr) | 137 | 159 | 0.98 | (0.77-1.25) | 0.88 | 0.90 |
| *ATM* | c.5071A>C (p.Ser1691Arg) | 162 | 221 | 0.87 | (0.70-1.07) | 0.19 | 0.039 |
| *ATM* | c.5558A>T (p.Asp1853Val) | 383 | 432 | 1.02 | (0.89-1.18) | 0.77 | 0.73 |
| *ATM* | c.6067G>A (p.Gly2023Arg) | 120 | 147 | 0.93 | (0.72-1.19) | 0.55 | 0.53 |
| *ATM* | c.6313A>G (p.Arg2105Gly) | 128 | 130 | 1.17 | (0.91-1.50) | 0.22 | 0.10 |
| *BRCA1* | c.2521C>T (p.Arg841Trp) | 96 | 165 | 0.67 | (0.52-0.87) | 0.0027 | 0.0016 |
| *BRCA1* | c.2566T>C (p.Tyr856His) | 141 | 138 | 0.98 | (0.77-1.25) | 0.88 | 0.85 |
| *BRCA1* | c.3119G>A (p.Ser1040Asn) | 973 | 1083 | 0.99 | (0.90-1.09) | 0.86 | 0.44 |
| *BRCA1* | c.4039A>G (p.Arg1347Gly) | 330 | 391 | 0.95 | (0.81-1.10) | 0.48 | 0.73 |
| *BRCA1* | c.4535G>T (p.Ser1512Ile) | 238 | 310 | 0.93 | (0.78-1.11) | 0.43 | 0.45 |
| *BRCA1* | c.4956G>A (p.Met1652Ile) | 1075 | 1117 | 1.06 | (0.97-1.16) | 0.21 | 0.48 |
| *BRCA2* | c.125A>G (p.Tyr42Cys) | 101 | 109 | 1.03 | (0.78-1.37) | 0.82 | 0.81 |
| *BRCA2* | c.865A>C (p.Asn289His) | 2166 | 2235 | 1.06 | (1.00-1.13) | 0.068 | 0.049 |
| *BRCA2* | c.978C>A (p.Ser326Arg) | 95 | 96 | 1.05 | (0.79-1.41) | 0.73 | 0.54 |
| *BRCA2* | c.1151C>T (p.Ser384Phe) | 76 | 97 | 0.87 | (0.63-1.18) | 0.37 | 0.42 |
| *BRCA2* | c.1792A>G (p.Thr598Ala) | 147 | 155 | 1.03 | (0.81-1.30) | 0.80 | 0.54 |
| *BRCA2* | c.2971A>G (p.Asn991Asp) | 2198 | 2279 | 1.06 | (0.99-1.13) | 0.087 | 0.090 |
| *BRCA2* | c.4258G>T (p.Asp1420Tyr) | 426 | 418 | 1.14 | (0.99-1.32) | 0.060 | 0.11 |
| *BRCA2* | c.5744C>T (p.Thr1915Met) | 1461 | 1675 | 0.97 | (0.90-1.05) | 0.44 | 0.30 |
| *BRCA2* | c.5785A>G (p.Ile1929Val) | 79 | 71 | 1.00 | (0.72-1.39) | 0.98 | 0.96 |
| *BRCA2* | c.6100C>T (p.Arg2034Cys) | 301 | 309 | 1.06 | (0.90-1.26) | 0.47 | 0.35 |
| *BRCA2* | c.8149G>T (p.Ala2717Ser) | 98 | 130 | 0.84 | (0.64-1.10) | 0.21 | 0.15 |
| *BRCA2* | c.8182G>A (p.Val2728Ile) | 219 | 208 | 1.25 | (1.02-1.53) | 0.030 | 0.17 |
| *BRCA2* | c.8187G>T (p.Lys2729Asn) | 67 | 60 | 1.03 | (0.72-1.47) | 0.86 | 0.63 |
| *BRCA2* | c.8567A>C (p.Glu2856Ala) | 133 | 172 | 0.89 | (0.70-1.13) | 0.32 | 0.56 |
| *BRCA2* | c.8851G>A (p.Ala2951Thr) | 299 | 340 | 1.04 | (0.88-1.23) | 0.62 | 0.24 |
| *BRCA2* | c.10234A>G (p.Ile3412Val) | 272 | 280 | 0.95 | (0.80-1.13) | 0.58 | 0.36 |
| *CHEK2* | c.470T>C (p.Ile157Thr) | 520 | 438 | 1.24 | (1.09-1.42) | 0.0013 | 0.0028 |
| *CHEK2* | c.538C>T (p.Arg180Cys) | 140 | 124 | 1.44 | (1.12-1.84) | 0.0040 | 0.0016 |
| *PALB2* | c.925A>G (p.Ile309Val) | 89 | 63 | 1.33 | (0.95-1.84) | 0.092 | 0.42 |
| *PALB2* | c.1010T>C (p.Leu337Ser) | 1120 | 1232 | 0.93 | (0.86-1.02) | 0.11 | 0.58 |
| *PALB2* | c.2014G>C (p.Glu672Gln) | 1756 | 2027 | 0.96 | (0.89-1.02) | 0.19 | 0.12 |
| *PALB2* | c.2590C>T (p.Pro864Ser) | 103 | 163 | 0.88 | (0.68-1.14) | 0.33 | 0.40 |
| *PALB2* | c.2794G>A (p.Val932Met) | 437 | 476 | 1.06 | (0.92-1.21) | 0.42 | 0.82 |
| *PALB2* | c.2816T>G (p.Leu939Trp) | 89 | 100 | 1.03 | (0.77-1.39) | 0.84 | 0.25 |
| *PALB2* | c.2993G>A (p.Gly998Glu) | 1307 | 1529 | 0.95 | (0.87-1.02) | 0.16 | 0.084 |

^a^ P-value for association in all training samples

**Table S13:** P-values for goodness-of-fit tests performed in validation dataset.

| Gene | Chi-squared test *P*-value | | |
| --- | --- | --- | --- |
|  | Logistic regression model^†^ | Mixture model with fixed α^a^ | Mixture model with fixed variant probabilities^b^ |
| *ATM* | 0.28 | 0.56 | 0.69 |
| *BRCA1* | 0.74 | 0.43 | 0.40 |
| *BRCA2* | 0.77 | 0.56 | 0.48 |
| *CHEK2* | 0.79 | 0.76 | 0.85 |
| *PALB2* | 0.87 | 0.76 | 0.68 |

*P*-value from chi-squared test comparing expected and observed numbers of cases and controls by risk category^a^ or posterior probability interval^b^ in the validation data set

**Additional Note: Breast Cancer Association Consortium funding and acknowledgements, and additional references.**

**Funding**

A.B Spurdle, M.T. Parsons and C. Fortuno were supported by funding from the Australian National Medical Research Council (IDs 1177524, 1101400, 1161589). The **ABCS** study was supported by the Dutch Cancer Society [grants NKI 2007-3839; 2009 4363]. The **ACP** study is funded by the Breast Cancer Research Trust, UK. KM and AL are supported by the NIHR Manchester Biomedical Research Centre, the Allan Turing Institute under the EPSRC grant EP/N510129/1. The work of the **BBCC** was partly funded by ELAN-Fond of the University Hospital of Erlangen. For **BIGGS**, ES is supported by NIHR Comprehensive Biomedical Research Centre, Guy's & St. Thomas' NHS Foundation Trust in partnership with King's College London, United Kingdom. IT is supported by the Oxford Biomedical Research Centre. The BREast Oncology GAlician Network (**BREOGAN**) is funded by Acción Estratégica de Salud del Instituto de Salud Carlos III FIS PI12/02125/Cofinanciado and FEDER PI17/00918/Cofinanciado FEDER; Acción Estratégica de Salud del Instituto de Salud Carlos III FIS Intrasalud (PI13/01136); Programa Grupos Emergentes, Cancer Genetics Unit, Instituto de Investigacion Biomedica Galicia Sur. Xerencia de Xestion Integrada de Vigo-SERGAS, Instituto de Salud Carlos III, Spain; Grant 10CSA012E, Consellería de Industria Programa Sectorial de Investigación Aplicada, PEME I + D e I + D Suma del Plan Gallego de Investigación, Desarrollo e Innovación Tecnológica de la Consellería de Industria de la Xunta de Galicia, Spain; Grant EC11-192. Fomento de la Investigación Clínica Independiente, Ministerio de Sanidad, Servicios Sociales e Igualdad, Spain; and Grant FEDER-Innterconecta. Ministerio de Economia y Competitividad, Xunta de Galicia, Spain. The **BSUCH** study was supported by the Dietmar-Hopp Foundation, the Helmholtz Society and the German Cancer Research Center (DKFZ). **CCGP** is supported by funding from the University of Crete. The **CECILE** study was supported by Fondation de France, Institut National du Cancer (INCa), Ligue Nationale contre le Cancer, Agence Nationale de Sécurité Sanitaire, de l'Alimentation, de l'Environnement et du Travail (ANSES), Agence Nationale de la Recherche (ANR). The **CGPS** was supported by the Chief Physician Johan Boserup and Lise Boserup Fund, the Danish Medical Research Council, and Herlev and Gentofte Hospital. The **CNIO-BCS** was supported by the Instituto de Salud Carlos III, the Red Temática de Investigación Cooperativa en Cáncer and grants from the Asociación Española Contra el Cáncer and the Fondo de Investigación Sanitario (PI11/00923 and PI12/00070). **COLBCCC** is supported by the German Cancer Research Center (DKFZ), Heidelberg, Germany. Diana Torres was in part supported by a postdoctoral fellowship from the Alexander von Humboldt Foundation. **FHRISK** and **PROCAS** are funded from NIHR grant PGfAR 0707-10031. DGE, AH and WGN are supported by the NIHR Manchester Biomedical Research Centre (IS-BRC-1215-20007). The **GC-HBOC** (German Consortium of Hereditary Breast and Ovarian Cancer) is supported by the German Cancer Aid (grant no 110837, coordinator: Rita K. Schmutzler, Cologne) and the Federal Ministry of Education and Research, Germany (grant no 01GY1901). This work was also funded by the European Regional Development Fund and Free State of Saxony, Germany (LIFE - Leipzig Research Centre for Civilization Diseases, project numbers 713-241202, 713-241202, 14505/2470, 14575/2470). The **GENICA** was funded by the Federal Ministry of Education and Research (BMBF) Germany grants 01KW9975/5, 01KW9976/8, 01KW9977/0 and 01KW0114, the Robert Bosch Foundation, Stuttgart, Deutsches Krebsforschungszentrum (DKFZ), Heidelberg, the Institute for Prevention and Occupational Medicine of the German Social Accident Insurance, Institute of the Ruhr University Bochum (IPA), Bochum, as well as the Department of Internal Medicine, Johanniter GmbH Bonn, Johanniter Krankenhaus, Bonn, Germany. Generation Scotland (**GENSCOT**) received core support from the Chief Scientist Office of the Scottish Government Health Directorates [CZD/16/6] and the Scottish Funding Council [HR03006]. Genotyping of the GS:SFHS samples was carried out by the Genetics Core Laboratory at the Edinburgh Clinical Research Facility, University of Edinburgh, Scotland and was funded by the Medical Research Council UK and the Wellcome Trust (Wellcome Trust Strategic Award “STratifying Resilience and Depression Longitudinally” (STRADL) Reference 104036/Z/14/Z). Funding for identification of cases and contribution to BCAC funded in part by the Wellcome Trust Seed Award “Temporal trends in incidence and mortality of molecular subtypes of breast cancer to inform public health, policy and prevention” Reference 207800/Z/17/Z. The **GESBC** was supported by the Deutsche Krebshilfe e. V. [70492] and the German Cancer Research Center (DKFZ). The **HABCS** study was supported by the Claudia von Schilling Foundation for Breast Cancer Research, by the Lower Saxonian Cancer Society, and by the Rudolf Bartling Foundation. The **HEBCS** was financially supported by the Helsinki University Hospital Research Fund, the Sigrid Juselius Foundation and The Cancer Foundation Finland. The **HEBON** study is supported by the Dutch Cancer Society grants NKI1998-1854, NKI2004-3088, NKI2007-3756, the Netherlands Organisation of Scientific Research grant NWO 91109024, the Pink Ribbon grants 110005 and 2014-187.WO76, the BBMRI grant NWO 184.021.007/CP46 and the Transcan grant JTC 2012 Cancer 12-054. The **HMBCS** was supported by a grant from the Friends of Hannover Medical School and by the Rudolf Bartling Foundation. The **HUBCS** was supported by a grant from the German Federal Ministry of Research and Education (RUS08/017), B.M. was supported by grant 17-44-020498, 17-29-06014 of the Russian Foundation for Basic Research, D.P. was supported by grant 18-29-09129 of the Russian Foundation for Basic Research, E.K was supported by the program for support the bioresource collections №007-030164/2 and by the megagrant from the Government of Russian Federation No. 075-15-2021-595, and the study was performed as part of the assignment of the Ministry of Science and Higher Education of the Russian Federation (№АААА-А16-116020350032-1). Financial support for **KARBAC** was provided through the regional agreement on medical training and clinical research (ALF) between Stockholm County Council and Karolinska Institutet, the Swedish Cancer Society, The Gustav V Jubilee foundation and Bert von Kantzows foundation. The **KARMA** study was supported by Märit and Hans Rausings Initiative Against Breast Cancer. The **KBCP** was financially supported by the special Government Funding (VTR) of Kuopio University Hospital grants, Cancer Fund of North Savo, the Finnish Cancer Organizations, and by the strategic funding of the University of Eastern Finland. **kConFab** is supported by a grant from the National Breast Cancer Foundation, and previously by the National Health and Medical Research Council (NHMRC), the Queensland Cancer Fund, the Cancer Councils of New South Wales, Victoria, Tasmania and South Australia, and the Cancer Foundation of Western Australia. Financial support for the AOCS was provided by the United States Army Medical Research and Materiel Command [DAMD17-01-1-0729], Cancer Council Victoria, Queensland Cancer Fund, Cancer Council New South Wales, Cancer Council South Australia, The Cancer Foundation of Western Australia, Cancer Council Tasmania and the National Health and Medical Research Council of Australia (NHMRC; 400413, 400281, 199600). G.C.T. and P.W. are supported by the NHMRC. RB was a Cancer Institute NSW Clinical Research Fellow. The **KOHBRA** study was partially supported by a grant from the Korea Health Technology R&D Project through the Korea Health Industry Development Institute (KHIDI), and the National R&D Program for Cancer Control, Ministry of Health & Welfare, Republic of Korea (HI16C1127; 1020350; 1420190). The **MARIE** study was supported by the Deutsche Krebshilfe e.V. [70-2892-BR I, 106332, 108253, 108419, 110826, 110828], the Hamburg Cancer Society, the German Cancer Research Center (DKFZ) and the Federal Ministry of Education and Research (BMBF) Germany [01KH0402]. The **MASTOS** study was supported by “Cyprus Research Promotion Foundation” grants 0104/13 and 0104/17, and the Cyprus Institute of Neurology and Genetics. **MBCSG** is supported by grants from the Italian Association for Cancer Research (AIRC). The Melbourne Collaborative Cohort Study (**MCCS**) cohort recruitment was funded by VicHealth and Cancer Council Victoria. The MCCS was further augmented by Australian National Health and Medical Research Council grants 209057, 396414 and 1074383 and by infrastructure provided by Cancer Council Victoria. Cases and their vital status were ascertained through the Victorian Cancer Registry and the Australian Institute of Health and Welfare, including the National Death Index and the Australian Cancer Database. **MYBRCA** is funded by research grants from the Wellcome Trust (v203477/Z/16/Z), the Malaysian Ministry of Higher Education (UM.C/HlR/MOHE/06) and Cancer Research Malaysia. The **NBCS** has received funding from the K.G. Jebsen Centre for Breast Cancer Research; the Research Council of Norway grant 193387/V50 (to A-L Børresen-Dale and V.N. Kristensen) and grant 193387/H10 (to A-L Børresen-Dale and V.N. Kristensen), South Eastern Norway Health Authority (grant 39346 to A-L Børresen-Dale) and the Norwegian Cancer Society (to A-L Børresen-Dale and V.N. Kristensen). The Ontario Familial Breast Cancer Registry (**OFBCR**) was supported by grant U01CA164920 from the USA National Cancer Institute of the National Institutes of Health. The content of this manuscript does not necessarily reflect the views or policies of the National Cancer Institute or any of the collaborating centers in the Breast Cancer Family Registry (BCFR), nor does mention of trade names, commercial products, or organizations imply endorsement by the USA Government or the BCFR. The **ORIGO** study was supported by the Dutch Cancer Society (RUL 1997-1505) and the Biobanking and Biomolecular Resources Research Infrastructure (BBMRI-NL CP16). The **PBCS** was funded by Intramural Research Funds of the National Cancer Institute, Department of Health and Human Services, USA. Genotyping for PLCO was supported by the Intramural Research Program of the National Institutes of Health, NCI, Division of Cancer Epidemiology and Genetics. The **PLCO** is supported by the Intramural Research Program of the Division of Cancer Epidemiology and Genetics and supported by contracts from the Division of Cancer Prevention, National Cancer Institute, National Institutes of Health. The **RBCS** was funded by the Dutch Cancer Society (DDHK 2004-3124, DDHK 2009-4318). The **SASBAC** study was supported by funding from the Agency for Science, Technology and Research of Singapore (A*STAR), the US National Institute of Health (NIH) and the Susan G. Komen Breast Cancer Foundation. **SEARCH** is funded by Cancer Research UK [C490/A10124, C490/A16561] and supported by the UK National Institute for Health Research Biomedical Research Centre at the University of Cambridge. The University of Cambridge has received salary support for PDPP from the NHS in the East of England through the Clinical Academic Reserve. **SGBCC** is funded by the National Research Foundation Singapore (NRF-NRFF2017-02), NUS start-up Grant, National University Cancer Institute Singapore (NCIS) Centre Grant, Breast Cancer Prevention Programme, Asian Breast Cancer Research Fund and the NMRC Clinician Scientist Award (SI Category). Population-based controls were from the Multi-Ethnic Cohort (MEC) funded by grants from the Ministry of Health, Singapore, National University of Singapore and National University Health System, Singapore. **SKKDKFZS** is supported by the DKFZ. The **SZBCS** was supported by Grant PBZ_KBN_122/P05/2004 and the program of the Minister of Science and Higher Education under the name "Regional Initiative of Excellence" in 2019-2022 project number 002/RID/2018/19 amount of financing 12 000 000 PLN. **UBCS** was supported by funding from National Cancer Institute (NCI) grant R01 CA163353 (to N.J. Camp) and the Women’s Cancer Center at the Huntsman Cancer Institute (HCI). Data collection for UBCS was supported by the Utah Population Database (UPDB) and Utah Cancer Registry (UCR). The UPDB is supported by HCI (including the Huntsman Cancer Foundation), University of Utah program in Personalized Health and Center for Clinical and Translational Science, and NCI grant P30 CA2014. The UCR is funded by the NCI's SEER Program, Contract No. HHSN261201800016I, the US Center for Disease Control and Prevention's National Program of Cancer Registries, Cooperative Agreement No. NU58DP0063200, the University of Utah and Huntsman Cancer Foundation.

**Acknowledgements**

We thank all the individuals who took part in these studies and all the researchers, clinicians, technicians and administrative staff who have enabled this work to be carried out. **ABCS** thanks the Blood bank Sanquin, The Netherlands. The **ACP** study wishes to thank the participants in the Thai Breast Cancer study. Special thanks also go to the Thai Ministry of Public Health (MOPH), doctors and nurses who helped with the data collection process. Finally, the study would like to thank Dr Prat Boonyawongviroj, the former Permanent Secretary of MOPH and Dr Pornthep Siriwanarungsan, the former Department Director-General of Disease Control who have supported the study throughout. **BBCS** thanks Eileen Williams, Elaine Ryder-Mills, Kara Sargus. **BIGGS** thanks Niall McInerney, Gabrielle Colleran, Andrew Rowan, Angela Jones. The **BREOGAN** study would not have been possible without the contributions of the following: Manuela Gago-Dominguez, Jose Esteban Castelao, Angel Carracedo, Victor Muñoz Garzón, Alejandro Novo Domínguez, Maria Elena Martinez, Sara Miranda Ponte, Carmen Redondo Marey, Maite Peña Fernández, Manuel Enguix Castelo, Maria Torres, Manuel Calaza (BREOGAN), José Antúnez, Máximo Fraga and the staff of the Department of Pathology and Biobank of the University Hospital Complex of Santiago-CHUS, Instituto de Investigación Sanitaria de Santiago, IDIS, Xerencia de Xestion Integrada de Santiago-SERGAS; Joaquín González-Carreró and the staff of the Department of Pathology and Biobank of University Hospital Complex of Vigo, Instituto de Investigacion Biomedica Galicia Sur, SERGAS, Vigo, Spain. The **BSUCH** study acknowledges the Principal Investigator, Barbara Burwinkel, and, thanks Peter Bugert, Medical Faculty Mannheim. **CCGP** thanks Styliani Apostolaki, Anna Margiolaki, Georgios Nintos, Maria Perraki, Georgia Saloustrou, Georgia Sevastaki, Konstantinos Pompodakis. **CGPS** thanks staff and participants of the Copenhagen General Population Study. For the excellent technical assistance: Dorthe Uldall Andersen, Maria Birna Arnadottir, Anne Bank, Dorthe Kjeldgård Hansen. The Danish Cancer Biobank is acknowledged for providing infrastructure for the collection of blood samples for the cases. **CNIO-BCS** thanks Guillermo Pita, Charo Alonso, Nuria Álvarez, Pilar Zamora, Primitiva Menendez, the Human Genotyping-CEGEN Unit (CNIO). **COLBCCC** thanks all patients, the physicians Justo G. Olaya, Mauricio Tawil, Lilian Torregrosa, Elias Quintero, Sebastian Quintero, Claudia Ramírez, José J. Caicedo, and Jose F. Robledo, and the technician Michael Gilbert for their contributions and commitment to this study. **FHRISK** and **PROCAS** thank NIHR for funding. The **GENICA** Network: Dr. Margarete Fischer-Bosch-Institute of Clinical Pharmacology, Stuttgart, and University of Tübingen, Germany [Hiltrud Brauch, Wing-Yee Lo, Reiner Hoppe], German Cancer Consortium (DKTK) and German Cancer Research Center (DKFZ), Partner Site Tübingen, 72074 Tübingen, Germany [Hiltrud Brauch], gefördert durch die Deutsche Forschungsgemeinschaft (DFG) im Rahmen der Exzellenzstrategie des Bundes und der Länder - EXC 2180 - 390900677 [Hiltrud Brauch], Department of Internal Medicine, Johanniter GmbH Bonn, Johanniter Krankenhaus, Bonn, Germany [Yon-Dschun Ko, Christian Baisch], Institute of Pathology, University of Bonn, Germany [Hans-Peter Fischer], Molecular Genetics of Breast Cancer, Deutsches Krebsforschungszentrum (DKFZ), Heidelberg, Germany [Ute Hamann], Institute for Prevention and Occupational Medicine of the German Social Accident Insurance, Institute of the Ruhr University Bochum (IPA), Bochum, Germany [Thomas Brüning, Beate Pesch, Sylvia Rabstein, Anne Lotz]; and Institute of Occupational Medicine and Maritime Medicine, University Medical Center Hamburg-Eppendorf, Germany [Volker Harth]. **HABCS** thanks Michael Bremer. **HEBCS** thanks Carl Blomqvist, Taru A. Muranen, Kristiina Aittomäki, Outi Malkavaara. **HEBON** Investigators are J. Margriet Collée, Frans B. L. Hogervorst, Maartje J. Hooning, Carolien M. Kets, Peter Devilee, Christi J. van Asperen, Matti A. Rookus, Marjanka K. Schmidt, Cora M. Aalfs, Muriel A. Adank, Margreet G. E. M. Ausems, Marinus J. Blok, Encarna B. Gómez Garcia, Bernadette A. M. Heemskerk-Gerritsen, Antoinette Hollestelle, Agnes Jager, Linetta B. Koppert, Marco Koudijs, Mieke Kriege, Hanne E. J. Meijers-Heijboer, Arjen R. Mensenkamp, Thea M. Mooij, Jan C. Oosterwijk, Ans M. W. van den Ouweland, Frederieke H. van der Baan, Annemieke H. van der Hout, Lizet E. van der Kolk, Rob B. van der Luijt, Carolien H. M. van Deurzen, Helena C. van Doorn, Klaartje van Engelen, Liselotte P. van Hest, Theo A. M. van Os, Senno Verhoef, Maartje J. Vogel & Juul T. Wijnen. **HMBCS** thanks Peter Hillemanns, Hans Christiansen and Johann H. Karstens. **HUBCS** thanks Darya Prokofyeva and Shamil Gantsev. **KARMA** and **SASBAC** thank the Swedish Medical Research Counsel. **KBCP** thanks Eija Myöhänen. **kConFab/AOCS** wish to thank Heather Thorne, Eveline Niedermayr, all the kConFab research nurses and staff, the heads and staff of the Family Cancer Clinics, and the Clinical Follow Up Study (which has received funding from the NHMRC, the National Breast Cancer Foundation, Cancer Australia, and the National Institute of Health (USA)) for their contributions to this resource, and the many families who contribute to kConFab. We thank all investigators of the **KOHBRA** (Korean Hereditary Breast Cancer) Study. **MARIE** thanks Petra Seibold, Nadia Obi, Sabine Behrens, Ursula Eilber and Muhabbet Celik. **MASTOS** thanks all the study participants and express appreciation to the doctors: Yiola Marcou, Eleni Kakouri, Panayiotis Papadopoulos, Simon Malas and Maria Daniel, as well as to all the nurses and volunteers who provided valuable help towards the recruitment of the study participants. **MBCSG** (Milan Breast Cancer Study Group): Manoukian Siranoush, Bernard Peissel, Jacopo Azzollini, Daniela Zaffaroni, Bernardo Bonanni, Irene Feroce, Mariarosaria Calvello, Aliana Guerrieri Gonzaga, Monica Marabelli, Davide Bondavalli and the personnel of the Cogentech Cancer Genetic Test Laboratory. The **MCCS** was made possible by the contribution of many people, including the original investigators, the teams that recruited the participants and continue working on follow-up, and the many thousands of Melbourne residents who continue to participate in the study. We thank the coordinators, the research staff and especially the **MYBRCA** thanks study participants and research staff (particularly Patsy Ng, Nurhidayu Hassan, Yoon Sook-Yee, Daphne Lee, Lee Sheau Yee, Phuah Sze Yee and Norhashimah Hassan) for their contributions and commitment to this study. The **OFBCR** thanks Teresa Selander, Nayana Weerasooriya and Steve Gallinger. **ORIGO** thanks E. Krol-Warmerdam, and J. Blom for patient accrual, administering questionnaires, and managing clinical information. The LUMC survival data were retrieved from the Leiden hospital-based cancer registry system (ONCDOC) with the help of Dr. J. Molenaar. **PBCS** thanks Louise Brinton, Mark Sherman, Neonila Szeszenia-Dabrowska, Beata Peplonska, Witold Zatonski, Pei Chao, Michael Stagner. We thank the **SEARCH** and **EPIC** teams. **SGBCC** thanks the participants and all research coordinators for their excellent help with recruitment, data and sample collection. **SKKDKFZ**S thanks all study participants, clinicians, family doctors, researchers and technicians for their contributions and commitment to this study. **SZBCS** thanks Ewa Putresza. **UBCS** thanks all study participants, the ascertainment, laboratory and research informatics teams at Huntsman Cancer Institute and Intermountain Healthcare, and Justin Williams, Brandt Jones, Melissa Cessna, Stacey Knight and Kerry Rowe for their important contributions to this study.

*kConFab Investigators*

Adrienne Sexton, Alex Dobrovic, Alice Christian, Alison Trainer, Allan Spigelman , Andrew Fellows, Andrew Shelling , Anna De Fazio, Anneke Blackburn, Ashley Crook, Bettina Meiser, Briony Patterson, Christine Clarke, Christobel Saunders, Clare Hunt, Clare Scott, David Amor, David Gallego Ortega, Deb Marsh, Edward Edkins, Elizabeth Salisbury, Eric Haan, Finlay Macrea, Gelareh Farshid, Geoff Lindeman, Georgia Trench, Graham Mann, Graham Giles, Grantley Gill, Heather Thorne, Ian Campbell, Ian Hickie, Liz Caldon, Ingrid Winship , James Cui, James Flanagan, James Kollias, Jane Visvader, Jennifer Stone, Jessica Taylor, Jo Burke, Jodi Saunus, John Forbes, John Hopper, Jonathan Beesley, Judy Kirk, Juliet French, Kathy Tucker, Kathy Wu, Kelly Phillips, Laura Forrest, Lara Lipton, Leslie Andrews, Lizz Lobb, Logan Walker, Maira Kentwell, Mandy Spurdle, Margaret Cummings, Margaret Gleeson, Marion Harris, Mark Jenkins, Mary Anne Young, Martin Delatycki, Mathew Wallis, Matthew Burgess, Melissa Brown, Melissa Southey, Michael Bogwitz , Michael Field, Michael Friedlander, Michael Gattas, Mona Saleh, Morteza Aghmesheh, Nick Hayward, Nick Pachter, Paul Cohen, Pascal Duijf, Paul James, Pete Simpson, Peter Fong, Phyllis Butow, Rachael Williams, Rick Kefford, Rodney Scott, Roger Milne, Rosemary Balleine, Sarah – Jane Dawson, Sheau Lok, Shona O'Connell, Sian Greening, Sophie Nightingale, Stacey Edwards, Stephen Fox, Sue-Anne McLachlan, Sunil Lakhani, Tracy Dudding, Yoland Antill**.**

*NBCS Collaborators*

Kristine K. Sahlberg, Anne-Lise Børresen-Dale, Inger Torhild Gram, Olav Engebråten, Bjørn Naume, Jürgen Geisler, OSBREAC, Grethe I. Grenaker Alnæs.

*SGBCC Investigators*

Swee Ho Lim, Ern Yu Tan, Benita Kiat Tee Tan, Su-Ming Tan, Veronique Kiak Mien Tan, Ching Wan Chan, Siau-Wei Tang, Celene Wei Qi Ng, Geok Hoon Lim, Jinnie Siyan Pang, Jung Ah Lee, Patrick Mun Yew Chan, Juliana Chen, Sarah Qinghui Lu, Yirong Sim, Wei Sean Yong, Preetha Madhukumar, Fuh Yong Wong, Joanne Yuen Yie Ngeow, Tira Jing Ying Tan, Wai Peng Lee, Chi Wei Mok, Chin Mui Seah, Linda Tan, E Shyong Tai, Xueling Sim, Peh Joo Ho, Alexis Jiaying Khng.

**Additional References**

1. Schmidt MK, Tollenaar RA, de Kemp SR, Broeks A, Cornelisse CJ, Smit VT, et al. Breast cancer survival and tumor characteristics in premenopausal women carrying the CHEK2*1100delC germline mutation. J Clin Oncol. 2007;25(1):64-9.

2. Michailidou K, Hall P, Gonzalez-Neira A, Ghoussaini M, Dennis J, Milne RL, et al. Large-scale genotyping identifies 41 new loci associated with breast cancer risk. Nat Genet. 2013;45(4):353-61, 61e1-2.

3. Schmidt MK, Hogervorst F, van Hien R, Cornelissen S, Broeks A, Adank MA, et al. Age- and Tumor Subtype-Specific Breast Cancer Risk Estimates for CHEK2*1100delC Carriers. J Clin Oncol. 2016;34(23):2750-60.

4. Fasching PA, Loehberg CR, Strissel PL, Lux MP, Bani MR, Schrauder M, et al. Single nucleotide polymorphisms of the aromatase gene (CYP19A1), HER2/neu status, and prognosis in breast cancer patients. Breast Cancer Res Treat. 2008;112(1):89-98.

5. Schrauder M, Frank S, Strissel PL, Lux MP, Bani MR, Rauh C, et al. Single nucleotide polymorphism D1853N of the ATM gene may alter the risk for breast cancer. J Cancer Res Clin Oncol. 2008;134(8):873-82.

6. Colleran G, McInerney N, Rowan A, Barclay E, Jones AM, Curran C, et al. The TGFBR1*6A/9A polymorphism is not associated with differential risk of breast cancer. Breast Cancer Res Treat. 2010;119(2):437-42.

7. McInerney N, Colleran G, Rowan A, Walther A, Barclay E, Spain S, et al. Low penetrance breast cancer predisposition SNPs are site specific. Breast Cancer Res Treat. 2009;117(1):151-9.

8. Jiang X, Castelao JE, Chavez-Uribe E, Fernandez Rodriguez B, Celeiro Munoz C, Redondo CM, et al. Family history and breast cancer hormone receptor status in a Spanish cohort. PLoS One. 2012;7(1):e29459.

9. Redondo CM, Gago-Dominguez M, Ponte SM, Castelo ME, Jiang X, Garcia AA, et al. Breast feeding, parity and breast cancer subtypes in a Spanish cohort. PLoS One. 2012;7(7):e40543.

10. Ali AM, Schmidt MK, Bolla MK, Wang Q, Gago-Dominguez M, Castelao JE, et al. Alcohol consumption and survival after a breast cancer diagnosis: a literature-based meta-analysis and collaborative analysis of data for 29,239 cases. Cancer Epidemiol Biomarkers Prev. 2014;23(6):934-45.

11. Cruz GI, Martinez ME, Natarajan L, Wertheim BC, Gago-Dominguez M, Bondy M, et al. Hypothesized role of pregnancy hormones on HER2+ breast tumor development. Breast Cancer Res Treat. 2013;137(1):237-46.

12. Gago-Dominguez M, Castelao JE, Gude F, Fernandez MP, Aguado-Barrera ME, Ponte SM, et al. Alcohol and breast cancer tumor subtypes in a Spanish Cohort. Springerplus. 2016;5:39.

13. Yang R, Dick M, Marme F, Schneeweiss A, Langheinz A, Hemminki K, et al. Genetic variants within miR-126 and miR-335 are not associated with breast cancer risk. Breast Cancer Res Treat. 2011;127(2):549-54.

14. Menegaux F, Truong T, Anger A, Cordina-Duverger E, Lamkarkach F, Arveux P, et al. Night work and breast cancer: a population-based case-control study in France (the CECILE study). Int J Cancer. 2013;132(4):924-31.

15. Weischer M, Bojesen SE, Tybjaerg-Hansen A, Axelsson CK, Nordestgaard BG. Increased risk of breast cancer associated with CHEK2*1100delC. J Clin Oncol. 2007;25(1):57-63.

16. Milne RL, Ribas G, Gonzalez-Neira A, Fagerholm R, Salas A, Gonzalez E, et al. ERCC4 associated with breast cancer risk: a two-stage case-control study using high-throughput genotyping. Cancer Res. 2006;66(19):9420-7.

17. Evans DG, Astley S, Stavrinos P, Harkness E, Donnelly LS, Dawe S, et al. Improvement in risk prediction, early detection and prevention of breast cancer in the NHS Breast Screening Programme and family history clinics: a dual cohort study. Programme Grants for Applied Research. Southampton (UK)2016.

18. Ingham SL, Warwick J, Buchan I, Sahin S, O'Hara C, Moran A, et al. Ovarian cancer among 8,005 women from a breast cancer family history clinic: no increased risk of invasive ovarian cancer in families testing negative for BRCA1 and BRCA2. J Med Genet. 2013;50(6):368-72.

19. Kast K, Rhiem K, Wappenschmidt B, Hahnen E, Hauke J, Bluemcke B, et al. Prevalence of BRCA1/2 germline mutations in 21 401 families with breast and ovarian cancer. J Med Genet. 2016;53(7):465-71.

20. Rhiem K, Engel C, Graeser M, Zachariae S, Kast K, Kiechle M, et al. The risk of contralateral breast cancer in patients from BRCA1/2 negative high risk families as compared to patients from BRCA1 or BRCA2 positive families: a retrospective cohort study. Breast Cancer Res. 2012;14(6):R156.

21. Graeser MK, Engel C, Rhiem K, Gadzicki D, Bick U, Kast K, et al. Contralateral breast cancer risk in BRCA1 and BRCA2 mutation carriers. J Clin Oncol. 2009;27(35):5887-92.

22. Engel C, Rhiem K, Hahnen E, Loibl S, Weber KE, Seiler S, et al. Prevalence of pathogenic BRCA1/2 germline mutations among 802 women with unilateral triple-negative breast cancer without family cancer history. BMC Cancer. 2018;18(1):265.

23. Pesch B, Ko Y, Brauch H, Hamann U, Harth V, Rabstein S, et al. Factors modifying the association between hormone-replacement therapy and breast cancer risk. Eur J Epidemiol. 2005;20(8):699-711.

24. Justenhoven C, Pierl CB, Haas S, Fischer HP, Baisch C, Hamann U, et al. The CYP1B1_1358_GG genotype is associated with estrogen receptor-negative breast cancer. Breast Cancer Res Treat. 2008;111(1):171-7.

25. Smith BH, Campbell A, Linksted P, Fitzpatrick B, Jackson C, Kerr SM, et al. Cohort Profile: Generation Scotland: Scottish Family Health Study (GS:SFHS). The study, its participants and their potential for genetic research on health and illness. Int J Epidemiol. 2013;42(3):689-700.

26. Chang-Claude J, Eby N, Kiechle M, Bastert G, Becher H. Breastfeeding and breast cancer risk by age 50 among women in Germany. Cancer Causes Control. 2000;11(8):687-95.

27. Dork T, Bendix R, Bremer M, Rades D, Klopper K, Nicke M, et al. Spectrum of ATM gene mutations in a hospital-based series of unselected breast cancer patients. Cancer Res. 2001;61(20):7608-15.

28. Syrjakoski K, Vahteristo P, Eerola H, Tamminen A, Kivinummi K, Sarantaus L, et al. Population-based study of BRCA1 and BRCA2 mutations in 1035 unselected Finnish breast cancer patients. J Natl Cancer Inst. 2000;92(18):1529-31.

29. Kilpivaara O, Bartkova J, Eerola H, Syrjakoski K, Vahteristo P, Lukas J, et al. Correlation of CHEK2 protein expression and c.1100delC mutation status with tumor characteristics among unselected breast cancer patients. Int J Cancer. 2005;113(4):575-80.

30. Fagerholm R, Hofstetter B, Tommiska J, Aaltonen K, Vrtel R, Syrjakoski K, et al. NAD(P)H:quinone oxidoreductase 1 NQO1*2 genotype (P187S) is a strong prognostic and predictive factor in breast cancer. Nat Genet. 2008;40(7):844-53.

31. Bogdanova N, Cybulski C, Bermisheva M, Datsyuk I, Yamini P, Hillemanns P, et al. A nonsense mutation (E1978X) in the ATM gene is associated with breast cancer. Breast Cancer Res Treat. 2009;118(1):207-11.

32. Wendt C, Lindblom A, Arver B, von Wachenfeldt A, Margolin S. Tumour spectrum in non-BRCA hereditary breast cancer families in Sweden. Hered Cancer Clin Pract. 2015;13(1):15.

33. Margolin S, Werelius B, Fornander T, Lindblom A. BRCA1 mutations in a population-based study of breast cancer in Stockholm County. Genet Test. 2004;8(2):127-32.

34. Hartikainen JM, Tuhkanen H, Kataja V, Dunning AM, Antoniou A, Smith P, et al. An autosome-wide scan for linkage disequilibrium-based association in sporadic breast cancer cases in eastern Finland: three candidate regions found. Cancer Epidemiol Biomarkers Prev. 2005;14(1):75-80.

35. Hartikainen JM, Tuhkanen H, Kataja V, Eskelinen M, Uusitupa M, Kosma VM, et al. Refinement of the 22q12-q13 breast cancer--associated region: evidence of TMPRSS6 as a candidate gene in an eastern Finnish population. Clin Cancer Res. 2006;12(5):1454-62.

36. Mann GJ, Thorne H, Balleine RL, Butow PN, Clarke CL, Edkins E, et al. Analysis of cancer risk and BRCA1 and BRCA2 mutation prevalence in the kConFab familial breast cancer resource. Breast Cancer Res. 2006;8(1):R12.

37. Beesley J, Jordan SJ, Spurdle AB, Song H, Ramus SJ, Kjaer SK, et al. Association between single-nucleotide polymorphisms in hormone metabolism and DNA repair genes and epithelial ovarian cancer: results from two Australian studies and an additional validation set. Cancer Epidemiol Biomarkers Prev. 2007;16(12):2557-65.

38. Han SA, Park SK, Ahn SH, Lee MH, Noh DY, Kim LS, et al. The Korean Hereditary Breast Cancer (KOHBRA) study: protocols and interim report. Clin Oncol (R Coll Radiol). 2011;23(7):434-41.

39. Flesch-Janys D, Slanger T, Mutschelknauss E, Kropp S, Obi N, Vettorazzi E, et al. Risk of different histological types of postmenopausal breast cancer by type and regimen of menopausal hormone therapy. Int J Cancer. 2008;123(4):933-41.

40. Hadjisavvas A, Loizidou MA, Middleton N, Michael T, Papachristoforou R, Kakouri E, et al. An investigation of breast cancer risk factors in Cyprus: a case control study. BMC Cancer. 2010;10:447.

41. De Vecchi G, Verderio P, Pizzamiglio S, Manoukian S, Barile M, Fortuzzi S, et al. Evidences for association of the CASP8 -652 6N del promoter polymorphism with age at diagnosis in familial breast cancer cases. Breast Cancer Res Treat. 2009;113(3):607-8.

42. Catucci I, Verderio P, Pizzamiglio S, Manoukian S, Peissel B, Barile M, et al. SNPs in ultraconserved elements and familial breast cancer risk. Carcinogenesis. 2009;30(3):544-5; author reply 6.

43. Giles GG, English DR. The Melbourne Collaborative Cohort Study. IARC Sci Publ. 2002;156:69-70.

44. Phuah SY, Looi LM, Hassan N, Rhodes A, Dean S, Taib NA, et al. Triple-negative breast cancer and PTEN (phosphatase and tensin homologue) loss are predictors of BRCA1 germline mutations in women with early-onset and familial breast cancer, but not in women with isolated late-onset breast cancer. Breast Cancer Res. 2012;14(6):R142.

45. Mariapun S, Ho WK, Kang PC, Li J, Lindstrom S, Yip CH, et al. Variants in 6q25.1 Are Associated with Mammographic Density in Malaysian Chinese Women. Cancer Epidemiol Biomarkers Prev. 2016;25(2):327-33.

46. Aure MR, Jernstrom S, Krohn M, Vollan HK, Due EU, Rodland E, et al. Integrated analysis reveals microRNA networks coordinately expressed with key proteins in breast cancer. Genome Med. 2015;7(1):21.

47. Fleischer T, Edvardsen H, Solvang HK, Daviaud C, Naume B, Borresen-Dale AL, et al. Integrated analysis of high-resolution DNA methylation profiles, gene expression, germline genotypes and clinical end points in breast cancer patients. Int J Cancer. 2014;134(11):2615-25.

48. Fleischer T, Frigessi A, Johnson KC, Edvardsen H, Touleimat N, Klajic J, et al. Genome-wide DNA methylation profiles in progression to in situ and invasive carcinoma of the breast with impact on gene transcription and prognosis. Genome Biol. 2014;15(8):435.

49. Quigley DA, Fiorito E, Nord S, Van Loo P, Alnaes GG, Fleischer T, et al. The 5p12 breast cancer susceptibility locus affects MRPS30 expression in estrogen-receptor positive tumors. Mol Oncol. 2014;8(2):273-84.

50. John EM, Hopper JL, Beck JC, Knight JA, Neuhausen SL, Senie RT, et al. The Breast Cancer Family Registry: an infrastructure for cooperative multinational, interdisciplinary and translational studies of the genetic epidemiology of breast cancer. Breast Cancer Res. 2004;6(4):R375-89.

51. de Bock GH, Schutte M, Krol-Warmerdam EM, Seynaeve C, Blom J, Brekelmans CT, et al. Tumour characteristics and prognosis of breast cancer patients carrying the germline CHEK2*1100delC variant. J Med Genet. 2004;41(10):731-5.

52. Huijts PE, Vreeswijk MP, Kroeze-Jansema KH, Jacobi CE, Seynaeve C, Krol-Warmerdam EM, et al. Clinical correlates of low-risk variants in FGFR2, TNRC9, MAP3K1, LSP1 and 8q24 in a Dutch cohort of incident breast cancer cases. Breast Cancer Res. 2007;9(6):R78.

53. Garcia-Closas M, Egan KM, Newcomb PA, Brinton LA, Titus-Ernstoff L, Chanock S, et al. Polymorphisms in DNA double-strand break repair genes and risk of breast cancer: two population-based studies in USA and Poland, and meta-analyses. Hum Genet. 2006;119(4):376-88.

54. Pfeiffer RM, Park Y, Kreimer AR, Lacey JV, Jr., Pee D, Greenlee RT, et al. Risk prediction for breast, endometrial, and ovarian cancer in white women aged 50 y or older: derivation and validation from population-based cohort studies. PLoS Med. 2013;10(7):e1001492.

55. Kriege M, Hollestelle A, Jager A, Huijts PE, Berns EM, Sieuwerts AM, et al. Survival and contralateral breast cancer in CHEK2 1100delC breast cancer patients: impact of adjuvant chemotherapy. Br J Cancer. 2014;111(5):1004-13.

56. Wedren S, Lovmar L, Humphreys K, Magnusson C, Melhus H, Syvanen AC, et al. Oestrogen receptor alpha gene haplotype and postmenopausal breast cancer risk: a case control study. Breast Cancer Res. 2004;6(4):R437-49.

57. Lesueur F, Pharoah PD, Laing S, Ahmed S, Jordan C, Smith PL, et al. Allelic association of the human homologue of the mouse modifier Ptprj with breast cancer. Hum Mol Genet. 2005;14(16):2349-56.

58. Stevens KN, Fredericksen Z, Vachon CM, Wang X, Margolin S, Lindblom A, et al. 19p13.1 is a triple-negative-specific breast cancer susceptibility locus. Cancer Res. 2012;72(7):1795-803.

59. Jakubowska A, Cybulski C, Szymanska A, Huzarski T, Byrski T, Gronwald J, et al. BARD1 and breast cancer in Poland. Breast Cancer Res Treat. 2008;107(1):119-22.

60. Jakubowska A, Jaworska K, Cybulski C, Janicka A, Szymanska-Pasternak J, Lener M, et al. Do BRCA1 modifiers also affect the risk of breast cancer in non-carriers? Eur J Cancer. 2009;45(5):837-42.

61. Cybulski C, Kluzniak W, Huzarski T, Wokolorczyk D, Kashyap A, Jakubowska A, et al. Clinical outcomes in women with breast cancer and a PALB2 mutation: a prospective cohort analysis. Lancet Oncol. 2015;16(6):638-44.

62. Cybulski C, Carrot-Zhang J, Kluzniak W, Rivera B, Kashyap A, Wokolorczyk D, et al. Germline RECQL mutations are associated with breast cancer susceptibility. Nat Genet. 2015;47(6):643-6.

63. Madsen MJ, Knight S, Sweeney C, Factor R, Salama M, Stijleman IJ, et al. Reparameterization of PAM50 Expression Identifies Novel Breast Tumor Dimensions and Leads to Discovery of a Genome-Wide Significant Breast Cancer Locus at 12q15. Cancer Epidemiol Biomarkers Prev. 2018;27(6):644-52.

64. Camp NJ, Parry M, Knight S, Abo R, Elliott G, Rigas SH, et al. Fine-mapping CASP8 risk variants in breast cancer. Cancer Epidemiol Biomarkers Prev. 2012;21(1):176-81.
